# The preference for sugar over sweetener depends on a gut sensor cell

**DOI:** 10.1038/s41593-021-00982-7

**Published:** 2022-01-13

**Authors:** Kelly L. Buchanan, Laura E. Rupprecht, M. Maya Kaelberer, Atharva Sahasrabudhe, Marguerita E. Klein, Jorge A. Villalobos, Winston W. Liu, Annabelle Yang, Justin Gelman, Seongjun Park, Polina Anikeeva, Diego V. Bohórquez

**Affiliations:** 1 Laboratory of Gut Brain Neurobiology, Duke University, Durham, NC USA; 2 Duke University School of Medicine, Durham, NC USA; 3 Department of Medicine, Duke University, Durham, NC USA; 4 McGovern Institute for Brain Research, Massachusetts Institute of Technology, Cambridge, MA USA; 5 Research Laboratory of Electronics, Massachusetts Institute of Technology, Cambridge, MA USA; 6 Department of Chemistry, Massachusetts Institute of Technology, Cambridge, MA USA; 7 Department of Neurobiology, Duke University, Durham, NC USA; 8 Trinity College of Arts & Sciences, Duke University, Durham, NC USA; 9 Department of Bio and Brain Engineering, Korea Advanced Institute of Science and Technology (KAIST), Daejeon, Republic of Korea; 10 Departments of Materials Science & Engineering and Brain & Cognitive Sciences, Massachusetts Institute of Technology, Cambridge, MA USA; 11 Duke Institute for Brain Sciences, Duke University, Durham, NC USA; 12 Present Address: MSRB-I, room 221A, 203 Research Drive, Durham, NC USA

**Keywords:** Cellular neuroscience, Sensory processing

## Abstract

Guided by gut sensory cues, humans and animals prefer nutritive sugars over non-caloric sweeteners, but how the gut steers such preferences remains unknown. In the intestine, neuropod cells synapse with vagal neurons to convey sugar stimuli to the brain within seconds. Here, we found that cholecystokinin (CCK)-labeled duodenal neuropod cells differentiate and transduce luminal stimuli from sweeteners and sugars to the vagus nerve using sweet taste receptors and sodium glucose transporters. The two stimulus types elicited distinct neural pathways: while sweetener stimulated purinergic neurotransmission, sugar stimulated glutamatergic neurotransmission. To probe the contribution of these cells to behavior, we developed optogenetics for the gut lumen by engineering a flexible fiberoptic. We showed that preference for sugar over sweetener in mice depends on neuropod cell glutamatergic signaling. By swiftly discerning the precise identity of nutrient stimuli, gut neuropod cells serve as the entry point to guide nutritive choices.

## Abstract (web summary)

Buchanan, Rupprecht, Kaelberer and colleagues show that the preference for sugar over sweetener in mice depends on gut neuropod cells. Akin to other sensor cells, neuropod cells swiftly communicate the precise identity of stimuli to drive food choices.

## Main

Both sugar and artificial sweeteners elicit a sweet taste, but sugar is preferred by animals and humans. Even mice lacking taste receptors can distinguish sugar from sweetener or water^1–3^. Although sensing sweetness depends on the tongue, flavor-conditioning tests show that the duodenum is needed to discern sugar from sweeteners.

Table sugar, or sucrose, is a disaccharide made of d-glucose and d-fructose. Unlike d-fructose or the sweetener sucralose, d-glucose conditions a robust preference when infused into the duodenal lumen^3–7^. In fact, animals with prior d-glucose exposure identify the sugar entering the intestine within minutes^8^. This ability to identify d-glucose vanishes when the small intestine is bypassed^9,10^, suggesting that the duodenal epithelium is where the ‘sugar transducer’ cell resides. But up until now, the identity of these cells has remained elusive because of the lack of tools to control gut sensory processing with temporal and spatial precision.

In other epithelial surfaces, electrically excitable cells use molecular receptors to detect and transduce sensory stimuli onto a cranial nerve to guide behavior. In the nose, for instance, olfactory receptor cells transduce odorant stimuli through glutamatergic synapses onto second-order mitral cells to assist the animal in distinguishing odors^11^. In the tongue, sweet, bitter or umami taste receptor cells form purinergic synapses with afferent nerve fibers to guide an animal in distinguishing tastants^12^. In the gut, this function seems to be performed by neuropod cells^13,14^.

Neuropod cells were first documented when enteroendocrine cells, known for their release of hormones such as CCK, were found to form synapses with underlying mucosal nerves^15,16^. In 2018, CCK-labeled duodenal neuropod cells were shown to form glutamatergic synapses with the vagus nerve^14^. These cells use the neurotransmitter glutamate to transduce a d-glucose stimulus from the gut to the brain in milliseconds (see video https://youtu.be/3v92lRNOdlA).

We hypothesized that duodenal neuropod cells discern nutritive sugars from non-caloric artificial sweeteners to guide the animal’s preference for sugar over sweetener.

## Results

### The vagus nerve responds to sugars and sweeteners

**Fig. 1:**
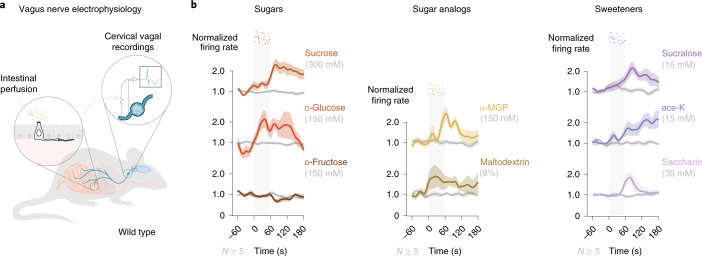
The vagus nerve responds to sugars and sweeteners. **a**, In anesthetized wild-type mice, stimuli were perfused through the duodenum from the pylorus to the ligament of Treitz, while electrical activity was recorded from the cervical vagus nerve. **b**, Vagal responses to intraduodenal stimuli, including baseline (PBS, gray traces), sucrose (300 mM) (*N* = 10), d-glucose (150 mM) (*N* = 5), d-fructose (150 mM) (*N* = 5), α-MGP (150 mM) (*N* = 8), maltodextrin (8%) (*N* = 5), sucralose (15 mM) (*N* = 11), acesulfame K (ace-K) (15 mM) (*N* = 5) and saccharin (30 mM) (*N* = 5), are shown. Peak responses and time to peak are quantified in Extended Data Fig. 1a,b. All peak responses except d-fructose were significant compared to baseline using a Kruskal–Wallis test with non-parametric comparisons using the Wilcoxon method. Gray vertical bars indicate infusion, the bold line indicates the mean, and shaded regions indicate s.e.m.

We first assessed whether a broad range of sugars, sugar analogs and non-caloric sweeteners perfused into the proximal small intestine would elicit rapid vagal responses. The vagus nerve responds to intraluminal sucrose^14,17^, but the response to other sugars and sweeteners commonly found in foods was unknown. Vagal firing rate was recorded in response to sugars (sucrose (300 mM), d-glucose (150 mM), d-fructose (150 mM) and d-galactose (150 mM)), sugar analogs (α-methylglucopyranoside (α-MGP; 150 mM) and maltodextrin (8%)) and sweeteners (sucralose (15 mM), acesulfame K (15 mM) and saccharin (30 mM)). All stimuli were perfused at physiological concentrations (see Methods)^14^ through the proximal duodenum, bypassing gustatory or gastric activation. Neural responses were recorded using electrodes placed at the cervical vagus nerve (Fig. 1a).

**Extended Data Fig. 1:**
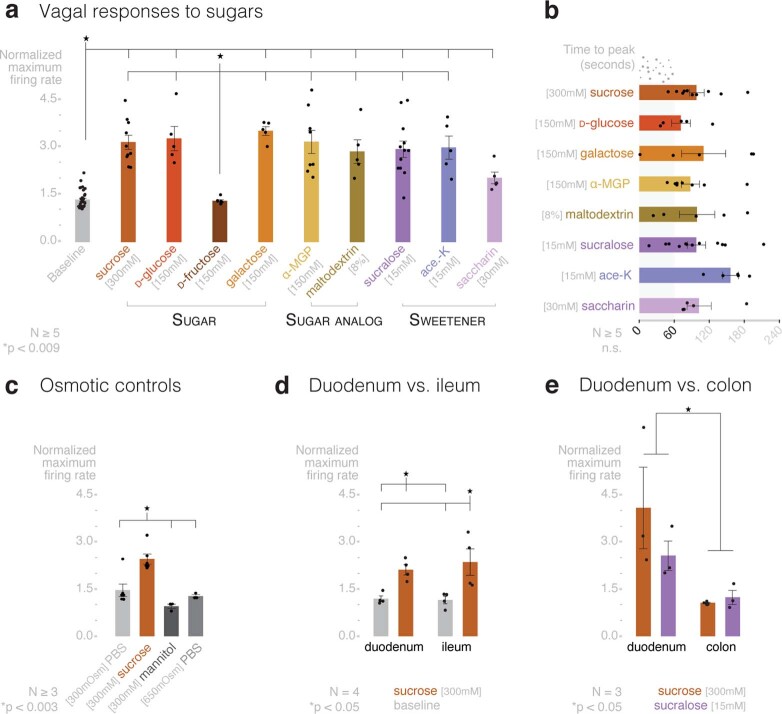
Vagal responses to sugars, sugar analogs, and sweeteners are not due to osmolarity and are specific to the small intestine, related to Fig. 1. **a**, Normalized maximum vagal firing rate to baseline (PBS), sugars (sucrose [300 mM] (N = 10), d-glucose [150 mM] (N = 5), d-fructose [150 mM] (N = 5), d-galactose [150 mM] (N = 5)), sugar analogs (alpha-methylglucopyranoside (α-MGP) [150 mM] (N = 8), and maltodextrin [8%] (N = 5)), and sweeteners (sucralose [15 mM] (N = 11), acesulfame K (ace-K) [15 mM] (N = 5), and saccharin [30 mM] (N = 5)). *p < 0.009 by Kruskal-Wallis test with non-parametric comparisons using Wilcoxon Method. P-values comparing baseline to each stimulus: sucrose, p < 0.0001; d-glucose, p = 0.0004; d-frucrose, p = 0.6868; d-galactose, p = 0.0004; α-MGP, p < 0.0001; maltodextrin, p = 0.0005; sucralose, p < 0.0001; ace-K, p = 0.0005; saccharin, p = 0.0008. **b**, Time-to-peak vagal firing rate for sugar stimuli (N as in **a**; n.s.). **c**, Normalized maximum vagal firing rate to intraduodenal sucrose [300 mM, ~650mOsm] (N = 6), mannitol [300 mM, ~650mOsm] (N = 3), and 2X PBS [650 mOsm] (N = 3). *p < 0.04 by Kruskal-Wallis test with non-parametric comparisons using Wilcoxon Method. **d**, Normalized maximum vagal firing rate to sucrose [300 mM] compared to baseline (PBS) infused into the duodenum (p = 0.0173) or ileum (p = 0.0036) (N = 4 mice per group; *p < 0.004, ANOVA with post hoc Tukey’s HSD test). **e**, Normalized maximum vagal firing rate to sucrose [300 mM] and sucralose [15 mM] infused intraluminally into the duodenum or proximal colon (N = 3 mice per group; *p = 0.0280, ANOVA with post hoc Tukey’s HSD test). Data are presented as mean values. Error bars = S.E.M.

Within seconds, almost all sugars elicited a significant increase in vagal firing rate (*N* ≥ 5 mice; *P* < 0.009 compared to baseline; Fig. 1b and Extended Data Fig. 1a,b). d-Fructose, however, did not elicit a vagal response. Unlike d-glucose, d-fructose diffuses passively through the epithelium and fails to condition a preference when infused into the intestine^18^. Control experiments showed that the vagal responses to sugars and sweeteners were not due to mechanical forces or osmolarity effects, as volume-matched normal PBS (200 μl), osmolarity-matched mannitol (650 mosM) or high-concentration PBS (650 mosM) did not elicit an increase in vagal firing (Extended Data Fig. 1c). Moreover, vagal responses to sugar and sweetener were confined to the small intestine. A vagal response to sucrose was only observed when infused into the duodenum and ileum but not the colon (Extended Data Fig. 1d,e). It is expected that glucose sensing occurs primarily in the proximal small intestine. This portion of the intestine is responsible for the postingestive rewarding effects of glucose^9,10^ and is where the vast majority of glucose is absorbed.

### The vagal response depends on duodenal neuropod cells

**Extended Data Fig. 2:**
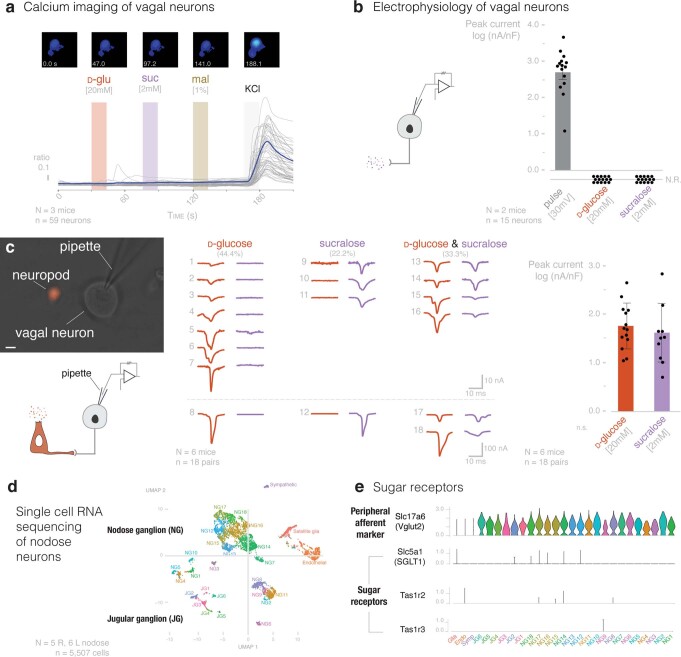
Vagal neuron response to sugars depends on intestinal Cck-labeled neuropod cells, related to Figs. 2 and 3. **a**, In wild-type vagal nodose neurons loaded with Fluo-4 and Fura Red, calcium activity was imaged in response to d-glucose [20 mM], sucralose [2 mM], maltodextrin [1%], and positive control KCl [50 mM] (N = 3 mice; n = 59 neurons). **b**, In CckCRE_tdTomato vagal nodose neurons cultured alone, current was recorded to a+40mV pulse, d-glucose [20 mM] stimulus, or sucralose [2 mM] stimulus (N = 2 mice; n = 15 neurons). No current response was observed to d-glucose or sucralose. Data are presented as mean values. Error bars = S.E.M. **c**, *Left*- Electrophysiology in co-cultures of vagal nodose neurons and CckCRE_tdTomato intestinal cells (bar = 10 μm). *Center-* Of 18 pairs of co-cultured neurons, excitatory post-synaptic potentials were recorded to d-glucose [20 mM] (44.4%), sucralose [2 mM] (22.2%), and both (33.3%) (N = 3 mice, n = 18 pairs). *Right* - Peak excitatory post-synaptic currents to d-glucose [20 mM] and sucralose [2 mM] (N = 3 mice; n = 18 pairs). Data are presented as mean values. Error bars = S.E.M. **d**, Single cell transcriptomic data projected onto the vagal nodose atlas^61^ showing 18 nodose ganglia (NG) and 6 jugular ganglia (JG) clusters (N = 5R, 6L nodose ganglia; n = 5,507 cells). **e**, Violin plots from single cell transcriptomic data showing transcripts for *Slc5a1* (SGLT1), *Tas1r2*, *Tas1r3*, and control *Slc17a6* (VGLUT2)—a peripheral afferent marker found ubiquitously in nodose and jugular ganglion neurons.

We hypothesized that the vagal responses depended on signals emanating from the intestinal epithelium. Vagal nodose neurons did not respond to sugars when isolated and cultured in vitro. Of all neurons imaged, 98.3% showed no calcium transients in response to d-glucose (20 mM), maltodextrin (1%) or sucralose (2 mM) (*N* = 3 mice, *n* = 59 neurons; viability confirmed by KCl (50 mM); Extended Data Fig. 2a). Therefore, we used optogenetics to test whether CCK-labeled neuropod cells enable transduction of sucrose, non-caloric sucralose and α-MGP stimuli. The sugar analog α-MGP is of interest because, like d-glucose, it is transported into the cell by the electrogenic sodium glucose transporter, but, unlike d-glucose, it is not further metabolized.

**Fig. 2:**
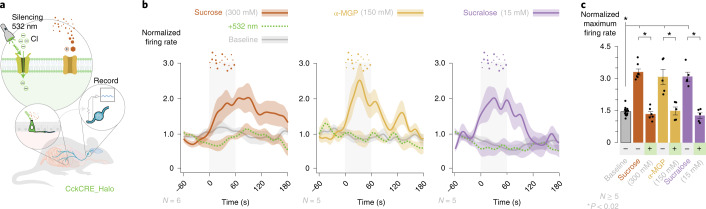
The vagal response to sugars, sugar analogs and non-caloric sweeteners depends on duodenal CCK-labeled neuropod cells. **a**, In CckCRE_Halo mice, vagal responses to intraduodenal stimuli were recorded, while CCK-labeled neuropod cells were simultaneously silenced with 532-nm light. **b**, Vagal responses to baseline (PBS), sucrose (300 mM) (*N* = 6), α-MGP (150 mM) (*N* = 5) and sucralose (15 mM) (*N* = 5) with and without intraluminal optical inhibition with 532-nm light. **c**, Quantification of peak responses (*P < 0.02 by Kruskal–Wallis test with non-parametric comparisons using Wilcoxon methods). Inhibition with 532-nm light significantly suppressed peak responses when delivered with intraluminal infusion of sucrose (*P* = 0.0034), α-MGP (*P* = 0.0122) and sucralose (*P* = 0.0122). In vitro coculture electrophysiology confirmed the dependence of vagal nodose neuron response to sugars on neuropod cells; see Extended Data Fig. 2c. Gray vertical bars indicate infusion, the bold line indicates the mean, and the shaded regions/error bars indicate s.e.m.

Using Cre/*loxP* recombination, we bred CckCRE_Halo mice in which the chloride pump halorhodopsin was expressed under the *Cck* promoter found in duodenal epithelial cells (Fig. 2a)^14,16^. When triggered by 532-nm light, halorhodopsin hyperpolarizes the cell membrane, silencing electrically excitable cells instantly. In CckCRE_Halo mice, vagal responses to luminal sucrose, α-MGP and sucralose remained unchanged in the presence of the control 473-nm light^14^. However, vagal responses to the same stimuli were completely abolished in the presence of silencing 532-nm light, indicating that vagal responses depend on duodenal CCK-labeled neuropod cells (*N* = 5–7 mice; *P* < 0.02 compared to response without laser; Fig. 2b,c).

In vitro, CCK-labeled neuropod cells synapse with nodose neurons to form connected pairs^14,16^. Thus, we used patch clamp electrophysiology in these cocultures to confirm the necessity of CCK-labeled neuropod cells in transducing sugar stimuli to vagal neurons in the absence of other cell types. In initial control experiments, we performed whole-cell patch clamp electrophysiology on cultured nodose neurons. Neither d-glucose (20 mM) nor sucralose (2 mM) elicited excitatory currents in nodose neurons when cultured alone (*N* = 2 mice, *n* = 15 neurons; Extended Data Fig. 2b). However, when nodose neurons were cocultured with duodenal CckCRE_tdTomato cells, we observed excitatory postsynaptic currents in connected neurons as follows: 44.4% to d-glucose only, 22.2% to sucralose only and 33.3% to both d-glucose and sucralose (*N* = 6 mice, *n* = 18 pairs; Extended Data Fig. 2c). Peak currents in the connected neurons were not statistically different between d-glucose and sucralose stimuli (Extended Data Fig. 2c). Thus, duodenal CCK-labeled neuropod cells are necessary to transduce luminal stimuli from both sugar and sweetener.

### Duodenal neuropod cells discern sugar from sweetener

**Fig. 3:**
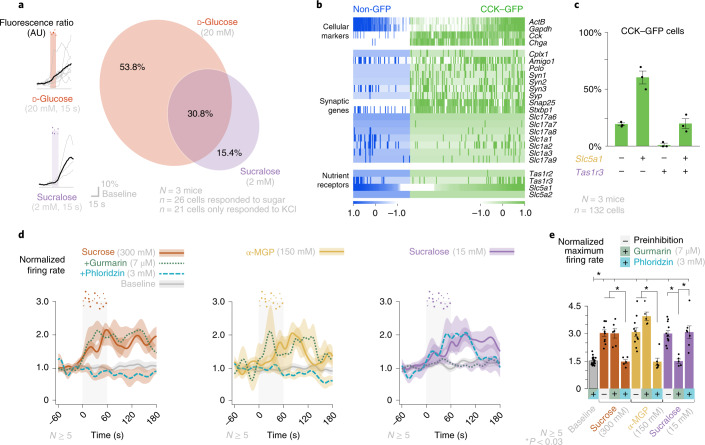
Duodenal neuropod cells discern sugar from sweetener. **a**, In CckCRE_tdTomato cells loaded with Fluo-4/Fura Red dye, calcium activity was imaged in response to d-glucose (20 mM) and sucralose (2 mM). Individual traces (left) and a Venn diagram illustrating overlap (right) are shown (*N* = 3 mice; *n* = 26 cells responded to d-glucose or sucralose, *n* = 21 cells responded to only KCl). No vagal neurons responded to stimuli, as shown in Extended Data Fig. 2a,b; F-Ratio, fluorescence intensity ratio of Fluo-4 divided by Fura Red. AU, arbitrary units. **b**, Heat map of gene expression in CCK–GFP and non-GFP intestinal epithelial cells by single-cell real time quantitative PCR (single cell RT–qPCR). Compared to non-GFP cells (*n* = 66), CCK–GFP cells (*n* = 132) overexpress genes associated with synapse formation (*Amigo1*, *Pclo*, *Syn1*–*Syn3*) and genes associated with vesicular function/release (*Cplx1*, *Syp*, *Snap25*, *Stxbp1*) (*N* = 3 mice; fold changes and *P* values are shown in Extended Data Fig. 3d). **c**, Of 132 CCK–GFP cells, 19.1 ± 1.2% express transcripts for neither *Slc5a1* (SGLT1) nor *Tas1r3* (T1R3), 60.1 ± 5.7% for only *Slc5a1*, 1.2 ± 1.2% for only *Tas1r3* and 19.6 ± 4.3% for both (*N* = 3 mice). **d**, Vagal responses to baseline (PBS) and stimuli perfused with and without the SGLT1 inhibitor phloridzin (3 mM) (sucrose (300 mM) *N* = 5, α-MGP (150 mM) *N* = 6, sucralose (15 mM) *N* = 7) or sweet taste inhibitor gurmarin (7 μM) (sucrose (300 mM) *N* = 6, α-MGP (150 mM) *N* = 5, sucralose (15 mM) *N* = 5). **e**, Quantification of peak vagal responses (*P < 0.03 by Kruskal–Wallis test with non-parametric comparisons using the Wilcoxon method). Phloridzin suppressed peak responses to sucrose (*P* = 0.0122) and α-MGP (*P* = 0.0131) but not sucralose (*P* = 0.5229). Gurmarin suppressed peak responses to sucralose only (*P* = 0.0122). Gray vertical bars indicate the infusion period, the bold line indicates the mean, and shaded regions/error bars indicate s.e.m.

We next tested how individual CCK-labeled neuropod cells respond to sugar and sweetener. Calcium transients were imaged in individual neuropod cells labeled with tdTomato (CckCRE_tdTomato) using the calcium indicator dyes Fluo-4 and Fura Red. A positive response was defined as an increase in the fluorescence ratio (Fluo-4/Fura Red) by greater than 10% (see Methods). In the 26 cells that responded to at least one sugar, 53.8% of cells responded to d-glucose only (20 mM), 15.4% to sucralose only (2 mM) and 30.8% to both d-glucose and sucralose (*N* = 3 mice, *n* = 47 viable cells; viability confirmed with KCl (50 mM); Fig. 3a).

Single-cell RT–qPCR was then used to determine the expression of molecular receptors used to sense sugars and sweeteners on individual CCK-labeled neuropod cells. Intestinal epithelial cells absorb d-glucose after it is cleaved from sucrose through active transport mediated by SGLT1^19^. In addition, some intestinal epithelial cells also express sweet taste receptors^20^. Vagal neurons, however, do not express transcripts for these sugar receptors (Extended Data Fig. 2d,e). Although SGLT1 and sweet taste receptors are known to be expressed in intestinal epithelial cells^20,21^, the expression profile of these receptors on individual CCK-labeled neuropod cells is unknown. We collected the small intestinal epithelial layers of mice expressing green fluorescent protein (GFP) under the *Cck* promoter (CCK–GFP) and performed RT–qPCR on single cells.

**Extended Data Fig. 3:**
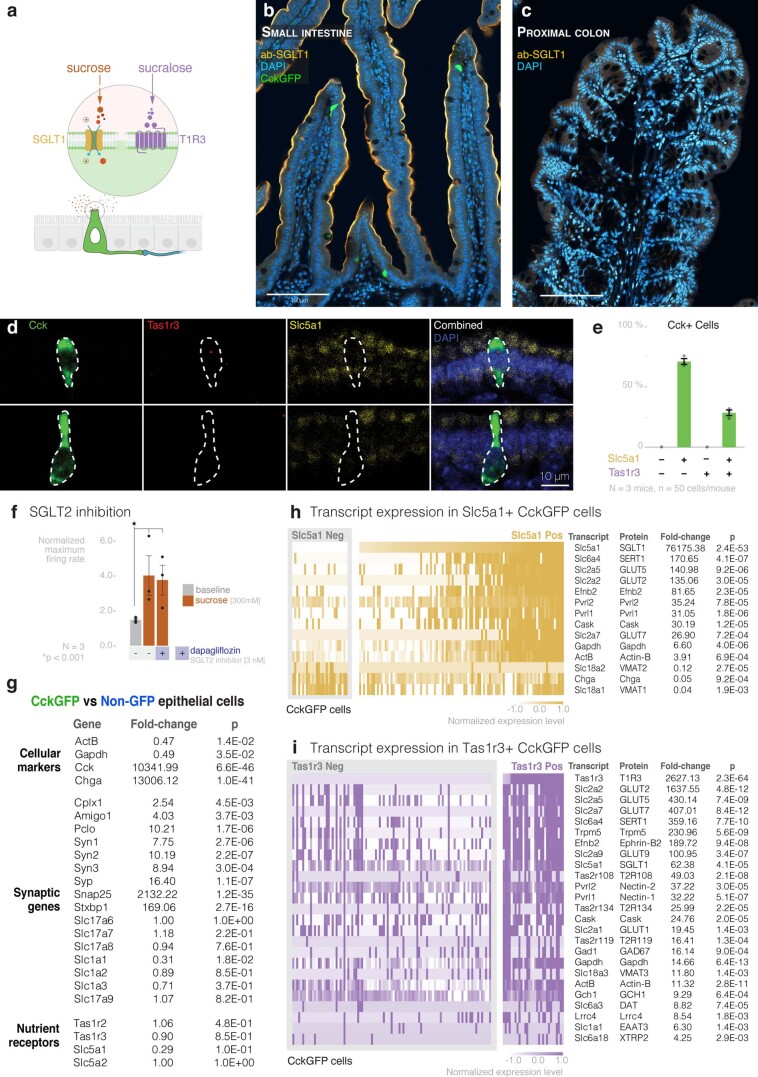
Cck-labeled neuropod cells express SGLT1 in the small intestine, related to Fig. 3. **a**, Cck-labeled neuropod cells express SGLT1 and T1R3. **b**, Immunofluorescent image of small intestine tissue stained with SGLT1 (yellow). Most small intestinal epithelial cells, that is absorptive enterocytes and Cck-labeled neuropod cells (green), express SGLT1. **c**, Immunofluorescent image of proximal colonic tissue stained with SGLT1 (yellow). Minimal SGLT1 staining was observed in the colon. **d**, Fluorescent in situ hybridization (FISH) images of Cck-labeled neuropod cells in duodenal tissue. *Top row*–cell with expression of *Cck*, *Tas1r3*, and *Slc5a1*. *Bottom row*–cell with expression of *Cck* and *Slc5a1*, but not *Tas1r3*. **e**, Quantification of FISH results. In accordance with the single-cell qPCR results (Figs. 3b,c), 71.3 ± 0.04% of CCK+ cells only expressed transcripts for *Slc5a1* while 28.7 ± 0.04% expressed transcripts for both *Slc5a1* and *Tas1r3* (N = 3 mice, n = 50 cells/mouse). Data are presented as mean values. Error bars = S.E.M. **f**, Normalized maximum vagal firing rate to baseline (PBS) and sucrose [300 mM] with and without SGLT2 inhibitor dapagliflozin [3 nM]. SGLT2 inhibition did not affect vagal firing in response to sucrose (N = 3 mice per group; *p = 0.0405 by ANOVA with post hoc Tukey’s HSD test). Data are presented as mean values. Error bars = S.E.M. **g**, Fold-change and p-values for genes shown in single cell qPCR heat map in Fig. 3b (N = 3 mice; n = 132 CckGFP + cells, n = 66 CckGFP- cells). **h-i**, Heat map of gene expression in CckGFP cells by single cell qRT-PCR. **h**, Genes significantly different between CckGFP cells positive and negative for *Slc5a1* (SGLT1) (N = 3 mice, n = 132 CckGFP cells, 104/132 *Slc5a1*+). **i**, Genes significantly different between CckGFP cells positive and negative for *Tas1r3* (T1R3) (N = 3 mice, n = 132 CckGFP cells, 31/132 *Tas1r3*+).

Compared to non-GFP cells, CCK–GFP cells were enriched in genes associated with synapse formation and vesicular function or release (*N* = 3 mice, *n* = 198 cells, 132 CCK–GFP^+^ compared to non-GFP cells; *q* value cutoff = 0.05 by two-tailed *t-*test; Fig. 3b; fold changes and *P* values for each gene are shown in Extended Data Fig. 3g). Moreover, individual CCK–GFP cells expressed SGLT1 and sweet taste receptors (Fig. 3b and Extended Data Fig. 3a). Immunohistochemistry was used to corroborate the presence of SGLT1 protein in the small intestine including CCK–GFP cells. Immunostaining of SGLT1 was prominent in the small intestine where sugars are absorbed compared to in the colon where minimal staining was observed (*N* = 3 mice; Extended Data Fig. 3b,c).

Receptor expression in individual CCK–GFP cells was as follows: *Tas1r2* was negligible, *Tas1r3* alone was in 1.2% (±1.2%) of cells, the SGLT1 transcript *Slc5a1* alone was in 60.1% (±5.7%) of cells and both *Tas1r3* and *Slc5a1* were in 19.6% (±4.3%) of cells (*N* = 3 mice, *n* = 132 CCK–GFP cells; Fig. 3c). These data were confirmed using fluorescence in situ hybridization in duodenal tissue from CCK–GFP mice, where *Slc5a1* alone was in 71.3% (±0.04%) of cells, and both *Slc5a1* and *Tas1r3* were in 28.7% (±0.04%) of cells (*N* = 3 mice, *n* = 50 cells per mouse; Extended Data Fig. 3d,e). Negligible transcript expression of *Tas1r2* suggests that T1R3 may function alone to detect sweet taste in CCK-labeled neuropod cells. While T1R2/T1R3 is the primary sensor of sweet stimuli in taste receptor cells, T1R3 homodimers in taste receptor cells can also detect sweet stimuli^22,23^. Other sensory epithelial cells, including GLP-1-secreting enteroendocrine cells^24^ and pancreatic beta cells^25^, have been shown to respond to sweet molecules using only T1R3.

We then determined if individual CCK–GFP cells with transcripts for SGLT1 (*Slc5a1*) and T1R3 (*Tas1r3*) also expressed synaptic transcripts, which is a distinctive feature of neuropod cells^14,16^. Compared to other CCK–GFP cells lacking the expression of *Slc5a1*, *Slc5a1-*expressing CCK–GFP cells had significantly increased expression of the presynaptic genes *Efnb2* (fold change of 81.6) and *Cask* (fold change of 30.2) and the synaptic adhesion genes *Pvrl1* (fold change of 31.05) and *Pvrl2* (fold change of 35.2) (*N* = 3 mice; *n* = 104 *Slc5a1*^+^CCK–GFP^+^ cells, *n* = 28 *Slc5a1*^*–*^CCK–GFP^+^ cells; *P* < 0.0001; Extended Data Fig. 3h). Compared to CCK–GFP cells lacking the expression of *Tas1r3*, *Tas1r3*-expressing CCK–GFP cells were also enriched in the expression of *Efnb2* (fold change of 189.7), *Cask* (fold change of 24.76), *Pvrl1* (fold change of 32.2) and *Pvrl2* (fold change of 37.2) (*N* = 3 mice; *n* = 31 *Tas1r3*^+^CCK–GFP^+^ cells, *n* = 101 *Tas1r3*^*–*^CCK–GFP^+^ cells; *P* < 0.0001; Extended Data Fig. 3i). These data show that CCK–GFP cells expressing *Slc5a1* and *Tas1r3* also express transcripts of proteins necessary for synaptic signaling.

Next, we assessed if the vagal responses to sugars or sweeteners were mediated by epithelial SGLTs or T1R3. When cleaved from sucrose, d-glucose enters the cell through SGLT1 for further metabolism. The synthetic analog α-MGP also enters the cell through SGLT1 but is not further metabolized. As such, its use allows for isolation of the entry of sugar into the cell through SGLT1. The results show that vagal responses to both sucrose and α-MGP are abolished when SGLTs are blocked with phloridzin (3 mM)^26^ (*N* ≥ 5 mice; *P* < 0.03 compared to preinhibition response; Fig. 3d,e). Phloridzin can also act at SGLT2, but inhibiting SGLT2 with dapagliflozin (3 nM) had no effect on the vagal response to sucrose (*N* = 3 mice; not significant compared to preinhibition response; Extended Data Fig. 3f). Single-cell RT–qPCR data showed no expression of the SGLT2 transcript *Slc5a2* in gut epithelial cells (Fig. 3b).

As expected, the SGLT inhibitor phloridzin did not affect the response to luminal sucralose (*N* ≥ 5 mice; not significant compared to preinhibition response; Fig. 3d,e). Instead, blocking sweet taste receptors, including T1R3, with gurmarin (7 μM)^27^ abolished the vagal response to sucralose (*N* ≥ 5 mice; *P* < 0.03 compared to preinhibition response; Fig. 3d,e). Gurmarin (7 μM), however, did not affect the response to sucrose (300 mM) or α-MGP (150 mM) (*N* ≥ 5 mice; not significant compared to preinhibition response; Fig. 3d,e). These data differ from the studies of taste transduction in the tongue, where both sucrose and sucralose activate T1R2/T1R3 receptors. In the gut, only sucralose elicited a taste receptor-mediated vagal response. The difference may be explained by the lack of expression of T1R2 in CCK-labeled neuropod cells and implies that T1R3 in the gut is more sensitive to sucralose than sucrose.

### Sugar, not sweetener, elicits glutamatergic neurotransmission

**Extended Data Fig. 4:**
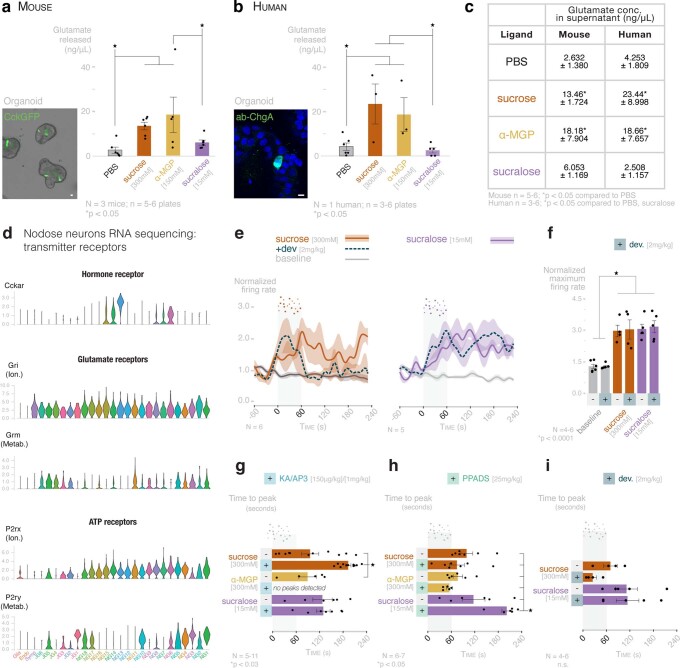
Cck-labeled neuropod cells use different neurotransmitters to distinguish sucrose from sucralose in both mouse and human, related to Fig. 4. **a-c**, Organoids cultured from mouse or human small intestinal tissue were stimulated with PBS, sucrose [300 mM], α-MGP [150 mM], and sucralose [15mM]. Glutamate in the supernatant was detected using a colorimetric assay. **a**, In CckGFP (green) intestinal organoids, sucrose and α-MGP elicited significant glutamate release compared to PBS control, while sucralose did not. **b**, Human duodenal organoids contain Chromogranin-A+ cells (ChgA, green)—a validated marker for enteroendocrine cells in human tissue that co-localizes with cholecystokinin^71^. Human duodenal organoids release glutamate to sucrose and α-MGP, but not to sucralose or PBS control. Bars=10 μm. **c**, Quantification of supernatant glutamate concentration from mouse and human organoids (mouse: N = 3 mice, n = 5-6 plates, *p < 0.05; human: N = 1 human sample, n = 3-6 plates, *p < 0.05). **d**, Violin plots from single cell transcriptomic data of nodose ganglia and jugular ganglia for *Cckar*, glutamate receptors (ionotropic (Ion.) and metabotropic (Metab.)), and ATP receptors (*P2rx* (ion.) and *P2ry* (metab.)) (N = 5 right, and 6 left murine nodose ganglia, n = 5,507 cells). **e**, Normalized vagal responses to baseline (PBS) and sucrose (left; N = 4) or sucralose (right; N = 6) before and after cholecystokinin-A receptor inhibition with devazepide [2mg/kg]. **f**, Quantification of peak vagal response from **(e)** *p < 0.0001. **g-i**, Time-to-peak vagal firing before and after inhibition of **(g)** glutamate receptors with KA/AP3 from Fig. 4b (*p < 0.03. p = 0.0031 comparing sucrose before and after KA/AP3); **(h)** P2 purinergic receptors with PPADS from Fig. 4d (*p < 0.05. p= 0.0369 comparing sucralose before and after PPADS); and (**i**) cholecystokinin-A receptors with devazepide from **(e)**. Gray vertical bars = infusion. Bold lines = mean, shaded regions/error bars = S.E.M. For vagal recordings, statistics by **(g)** Kruskal-Wallis test with non-parametric comparisons using Wilcoxon Method or **(f, h, i)** ANOVA with post hoc Tukey’s HSD test.

We then determined how duodenal CCK-labeled neuropod cells communicate different intestinal stimuli to the vagus nerve. d-Glucose stimulates individual neuropod cells to release glutamate^14^. CCK–GFP cells express transcripts for the vesicular glutamate transporters *Slc17a7* and *Slc17a8* and the synaptic glutamate transporters *Slc1a1*, *Slc1a2* and *Slc1a3* (Fig. 3b). Using intestinal organoids, we probed whether glutamate release is (1) specific to sugar and not sweetener and (2) conserved between mice and humans. Organoids were cultured from mouse proximal small intestine^28^ and human duodenum^29^. Sucrose (300 mM) and α-MGP (150 mM) elicited a significant release of glutamate compared to PBS (*N* = 3 mice, *n* = 5–6 assays in triplicate per stimuli, *P* < 0.05, Extended Data Fig. 4a,c; *N* = 1 human donor, *n* = 3–6 assays in triplicate per stimuli, *P* < 0.05, Extended Data Fig. 4b,c). By contrast, sucralose (15 mM) did not stimulate glutamate release (Extended Data Fig. 4a–c). These data suggest that glutamate release from the gut epithelium is specific to sugar and is conserved across mice and humans.

**Fig. 4:**
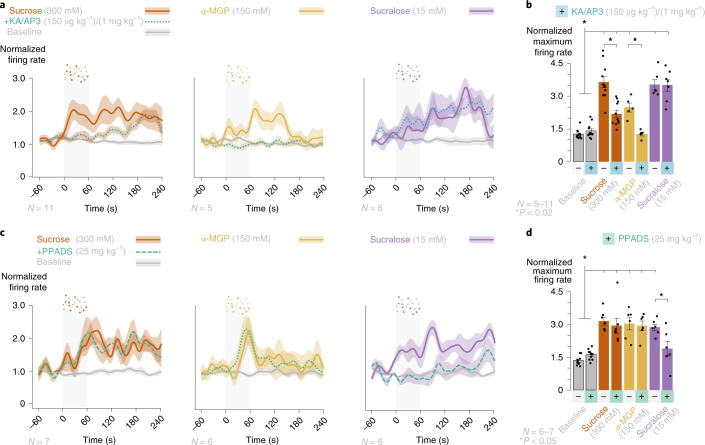
Sucrose and sucralose are transmitted to the vagus nerve by distinct neurotransmitters. **a**, Vagal responses to baseline (PBS), sucrose (300 mM), α-MGP (150 mM) and sucralose (15 mM) before and after inhibition of ionotropic/metabotropic glutamate receptors by KA (150 μg kg^–1^) with AP3 (1 mg kg^–1^). **b**, Quantification of peak responses to sucrose (*N* = 11), α-MGP (*N* = 5) and sucralose (*N* = 6) before and after glutamate receptor inhibition (*P < 0.02 by Kruskal–Wallis test with non-parametric comparisons using the Wilcoxon method). Glutamate receptor inhibitors significantly suppressed peak responses to sucrose (*P* = 0.0002) and α-MGP (*P* = 0.0122) but not sucralose (*P* = 0.9278). Time to peak vagal response is quantified in Extended Data Fig. 4g. **c**, Vagal responses to baseline, sucrose, α-MGP and sucralose before and after inhibition of P2 purinergic receptors with pyridoxalphosphate-6-azophenyl-2′,4′-disulfonic acid (PPADS; 25 mg kg^–1^). **d**, Quantification of peak responses to sucrose (*N* = 7), α-MGP (*N* = 6) and sucralose (*N* = 6) before and after purinergic receptor inhibition (*P < 0.05 by by Kruskal–Wallis test with non-parametric comparisons using the Wilcoxon method). PPADS significantly suppressed peak responses to sucralose (*P* = 0.0453) but not to sucrose (*P* = 0.1252) or α-MGP (*P* = 0.6889). Time to peak vagal response is quantified in Extended Data Fig. 4h. Gray vertical bars indicate infusion, the bold line indicates the mean, and shaded regions/error bars indicate s.e.m.

Transcripts for both metabotropic and ionotropic glutamate receptors are expressed in vagal nodose neurons (Extended Data Fig. 4d)^14^. Blocking ionotropic and metabotropic glutamate receptors with intraluminal perfusion of kynurenic acid (KA) (150 μg kg^–1^) plus l-(+)-2-amino-3-phosphonopropionic acid (AP3) (1 mg kg^–1^), respectively, decreased the early phase of the vagal response to sucrose and completely attenuated the response to α-MGP (*N* = 11 and 5; *P* < 0.02 compared to preinhibition response; Fig. 4a,b and Extended Data Fig. 4g). However, glutamatergic inhibition had no effect on the vagal response to sucralose (*N* = 6 mice; not significant compared to preinhibition response; Fig. 4a,b and Extended Data Fig. 4g). Notably, inhibiting glutamatergic neurotransmission eliminated the α-MGP response (Fig. 4a,b and Extended Data Fig. 4g). Therefore, the entry of sugar into the cell drives glutamatergic neurotransmission between neuropod cells and vagal neurons.

In addition to neurotransmission, there is a CCK hormonal signal emanating from duodenal CCK-labeled neuropod cells. Vagal neurons express the CCK-A receptor (Extended Data Fig. 4d). Therefore, we tested a potential role of CCK hormone in vagal responses to sucrose and sucralose. Blocking CCK-A receptors with devazepide (2 mg kg^–1^)^30^ blunted vagal response to sucrose 2 min after the onset of stimulus, leaving the first 120 s of the response intact. However, blocking CCK-A receptors did not affect the vagal response to sucralose (*N* = 5–6 mice; not significant compared to preinhibition response; Extended Data Fig. 4e,f,i). Therefore, the vagal response to sucralose was both glutamate and CCK independent.

We explored the possibility that sweetener may be transduced using a different neurotransmitter. In the tongue, taste receptor cells release ATP to activate purinergic receptors on sensory neurons in response to sweet stimuli^31^. Additionally, it has been shown that proglucagon-labeled enteroendocrine cells co-release hormone with the fast neurotransmitter ATP^32^. Thus, ATP was a candidate neurotransmitter for sucralose in the gut. We found that nodose neurons expressed purinergic receptors and that CCK–GFP cells expressed the vesicular nucleotide transporter for ATP *Slc17a9* (VNUT) (Fig. 3b and Extended Data Fig. 4d). Of all *Slc17a9*-expressing CCK–GFP cells, 31.1% (±8.2%) also expressed the sweet taste receptor transcript *Tas1r3*. Vagal responses to sucralose were also significantly attenuated by luminal inhibition of P2 purinergic receptors with PPADS (25 mg kg^–1^)^32^ (*N* = 6; *P* < 0.05 compared to preinhibition response; Fig. 4c,d and Extended Data Fig. 4h). The vagal response to sucrose and α-MGP were unchanged by purinergic receptor inhibition (*N* = 6–7; not significant compared to preinhibition response; Fig. 4c,d and Extended Data Fig. 4h). These data show that luminal transduction of sucralose onto the vagus nerve is through purinergic neurotransmission, while fast sucrose transduction is through glutamatergic neurotransmission.

**Extended Data Fig. 5:**
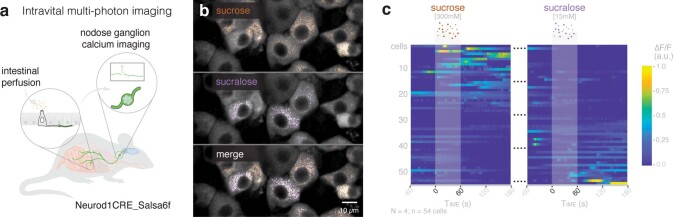
Distinct neuronal pathways transmit luminal sucrose and sucralose from gut to brain. **a**, In anesthetized Neurod1CRE_Salsa6f mice, right nodose ganglion neurons were imaged in vivo by intravital multi-photon calcium imaging while sugars were delivered from the pylorus to the ligament of Treitz. In these mice, nodose neurons express the calcium indicator GCaMP6f. **b**, Representative images of nodose ganglion neurons colored by response to sucrose (*top*) and sucralose (*middle*). Merged image (*bottom*) shows non-overlapping populations. **c**, Calcium activity was imaged in response to intraduodenal sucrose [300mM] then sucralose [15mM], or vice versa. Each row indicates one neuron’s response to both sucrose and sucralose (N = 4 mice, n = 54 cells).

We then asked whether sucrose and sucralose could activate distinct vagal neuron populations. Recent studies have used intravital two-photon imaging assays to show that individual vagal nodose neurons respond to different sensory modalities^17,33^. We used this assay in anesthetized animals expressing GCaMP6f in nodose neurons (see Methods). Vagal neurons were calcium imaged while sucrose (300 mM), sucralose (15 mM) or PBS were perfused directly into the duodenum to determine whether the same neuron or different neurons responded to both sugar and sweetener. The results showed that 40.7% of neurons responded to sucrose only, 22.2% to sucralose only and the remainder to neither stimulus (*N* = 4 mice, *n* = 54 neurons; Extended Data Fig. 5). Together, these findings show that duodenal neuropod cells use different neurotransmitters to convey stimuli from sucrose and sucralose onto distinct vagal nodose neuron populations.

### A flexible fiber for gut optogenetics

Now that it was established that neuropod cells discern sugar from sweetener, we sought to determine whether these epithelial transducers also guide the animal’s preference for sugar over sweetener. To test the contribution of these cells to behavior, a method was needed to silence neuropod cells while the mouse’s preference was recorded.

In the brain, the contribution of specific neurons to behaviors has been uncovered using optogenetics^34^. This technique relies on light-gated channels activated by laser light traditionally delivered using rigid silica fiberoptics. In the gut, however, we found that rigid fiberoptics puncture and perforate the intestinal wall. Recently, some efforts have been made to stimulate the outer muscular wall of the intestine^35^ or a small portion of the stomach^36^ in vivo. But no tool existed to control a specific population of gut epithelial cells diffused along several centimeters of the intestinal lumen in a living animal. As such, we developed a new device to deliver laser light into the gut lumen.

**Fig. 5:**
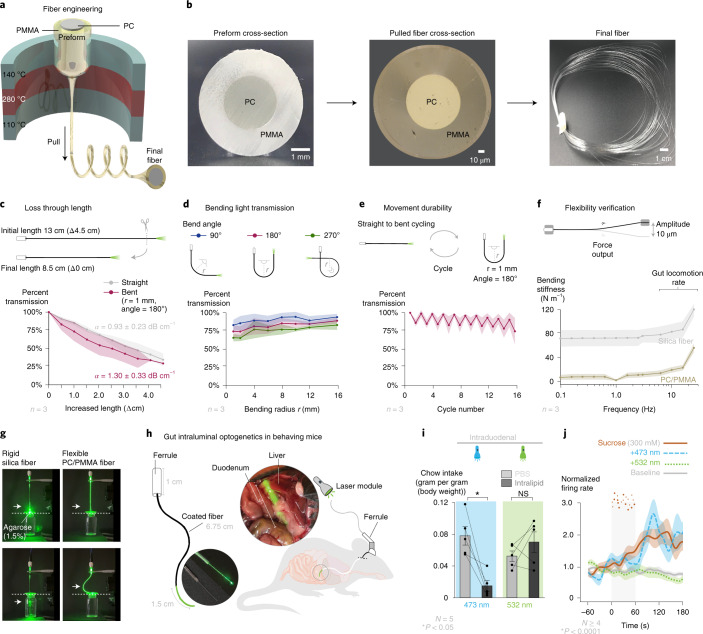
Development of a flexible fiberoptic device for optogenetic targeting of gut neuropod cells. **a**, Model of the thermal drawing process to obtain a flexible PC/PMMA fiber. **b**, Cross-section of the PC/PMMA preform (left), pulled PC/PMMA fiber (middle) and ~50-m fiber bundle (right). **c**, Light transmission for straight and bent flexible fibers using the cut-back method plotted as percentage of light output (*y* axis) from shortest length (∆0 cm). Loss coefficients (*α*) were determined as 0.93 dB cm^–1^ and 1.30 dB cm^–1^ for straight and bent fibers, respectively; *r*, radius. **d**, Light transmission for fibers bent at 90°, 180° and 270° at different radii of curvature (*x* axis) plotted as percentage of light output from a straight fiber (*y* axis). **e**, Light transmission for fibers during cyclic bending at 180° (odd cycles, straight; even, bent) plotted as percentage of light output from initial position (cycle = 0). **f**, The flexibility of silica and PC/PMMA fiber was measured by a dynamic mechanical analyzer at physiologic frequencies. For **c**–**f**, *n* = 3 fibers, the bold line indicates the mean, and the shaded regions indicate s.d. **g**, A conventional silica fiber pierces an agarose (1.5%) membrane, while the PC/PMMA flexible fiber bends and does not pierce the membrane. **h**, The flexible fiber was implanted into mice to target the lumen of the proximal duodenum. **i**, To validate the device in a known function of CCK-labeled neuropod cells, CckCRE_Halo mice received intragastric gavage of intralipid (7%, 0.1 ml per 10 g) with control 473-nm light, which reduced chow intake. This effect was reversed when CCK-labeled neuropod cells were silenced with 532-nm light (*N* = 5 mice; *P = 0.0193 by analysis of variance (ANOVA) with post hoc two-tailed paired Student’s *t*-test; error bars indicate s.e.m.); NS, not significant. **j**, To validate device longevity, vagal responses to baseline (PBS), sucrose without light, sucrose with control 473-nm light and sucrose with silencing 532-nm light were recorded in CckCRE_Halo mice 4 weeks after fiber implantation (*N* ≥ 4 mice per group; the bold line indicates the mean, and the shaded region indicates s.e.m.).

The system required a flexible fiberoptic with the following properties: (1) thin diameter for minimal footprint within the intestinal lumen, (2) low optical loss coefficient to deliver light to the gut lumen, (3) efficient light transmission even when bent and (4) durability for months when flexed inside the churning gut. First, we engineered a fiber preform of a poly-methyl methacrylate (PMMA) cladding layer around an optical core of polycarbonate (PC). Then, the preform was thermally drawn^37,38^ at 270 °C into a final flexible fiber 230 μm in diameter (Fig. 5a,b). To determine optical loss, the fiber was cut in 0.5-cm increments, and light transmission was measured when the fiber was either straight or bent to 180°. Percent transmission was compared to transmission at the shortest length. The loss coefficients were determined to be 0.93 dB cm^–1^ and 1.30 dB cm^–1^ for straight and bent fibers, respectively (Fig. 5c). Light transmission had minimal loss when bent at 90°, 180° and 270° angles compared to transmission when the fiber was held straight (Fig. 5d). Repeated 180° bending did not heavily influence light transmission (Fig. 5e). In addition, the device transmitted light with a 1.2-dB cm^–1^ loss and tolerated rapid bending at 10 Hz, which is above the physiological frequency of gut motility (Fig. 5f). Compared to rigid silica, the flexible fiberoptic did not pierce through a soft layer of 1.5% agarose, which is similar in consistency to the gut wall (Fig. 5g and Supplementary Video 1). The flexible fiberoptic was opacified to restrict light to the first 1.5 cm of the mouse small intestine (Fig. 5h).

To validate the device for gut optogenetics in freely moving mice, we investigated if silencing CCK-labeled neuropod cells eliminated the anorectic effect of a lipid gavage, an established physiological effect of CCK. A fat solution (intralipid, 7%) was delivered to CckCRE_Halo mice by gavage with simultaneous silencing (532-nm) or control (473-nm) light. Total food intake was significantly suppressed in mice with fat gavage and control light but not in mice with fat gavage and silencing light (Fig. 5i). We then confirmed the device’s durability by implanting it in the intestine of CckCRE_Halo mice. Four weeks later, control 473-nm light emitted from the device did not affect the vagal response to intraduodenal sucrose (Fig. 5j), whereas 532-nm light eliminated the response. These results corroborated the sustained functionality of the device to optogenetically modulate CCK-labeled neuropod cells in vivo.

### Sugar preference depends on duodenal neuropod cells

We then determined if CCK-labeled neuropod cells are necessary for mice to discern sucrose from sucralose. Mice were implanted with the flexible fiberoptic, acclimated to the phenotyping cage and tested for side preference. Each mouse was exposed to sucrose and sucralose until they demonstrated a stable preference for sucrose (see Methods). The location and power of the implanted device was corroborated at the end of the study. On each experimental day, implanted mice were given the choice between sucrose (300 mM) and sucralose (15 mM) for 1 h while receiving light stimulation to inhibit CCK-labeled neuropod cells (1 min on/2 min off, 5 V, 40 Hz, 20% duty cycle).

**Fig. 6:**
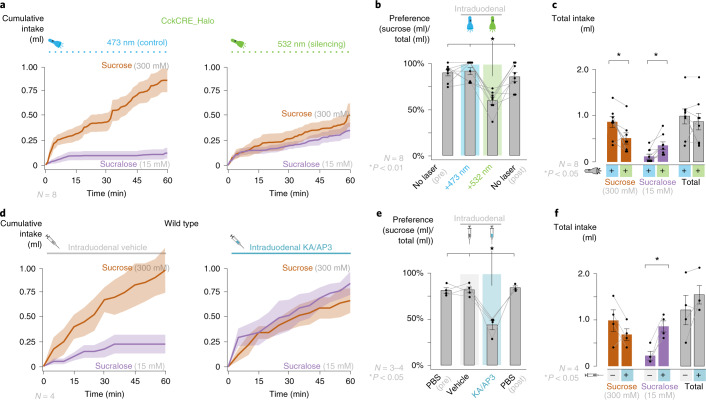
Sugar preference depends on duodenal neuropod cells. Mice with a stable preference for sucrose over sucralose chose between the two solutions during optogenetic (**a**–**c**) or pharmacologic (**d**–**f**) inhibition in a 1-h two-bottle choice assay. **a**, In CckCRE_Halo mice implanted with a flexible fiberoptic, average traces show sucrose and sucralose consumption in the presence of intraduodenal control 473-nm (left) or silencing 532-nm (right) light. For littermate controls, see Extended Data Fig. 6a–c. **b**, Quantification of preference at 1 h with no laser (pretest/posttest) and with control 473-nm light and silencing 532-nm light. Silencing 532-nm light significantly reduced sucrose preference compared to pretest (*P* = 0.0012), posttest (*P* = 0.0057) and control 473-nm light (*P* = 0.0003). **c**, Quantification of total intake during optogenetic silencing at 1 h. Silencing 532-nm light significantly decreased sucrose intake (*P* = 0.0224) and increased sucralose intake (*P* = 0.048) with no change in total intake (*P* = 0.4347). For **a**–**c**, *N* = 8 CckCRE_Halo mice; *P < 0.05 by repeated measures ANOVA with post hoc two-tailed paired *t*-test. **d**, In wild-type mice with intraduodenal catheters, average traces show sucrose and sucralose consumption in the presence of vehicle (PBS + NaOH, pH 7.4; left) or local dose of ionotropic/metabotropic glutamate receptor inhibitors KA/AP3 (15 ng per 0.1 μg in 0.4 ml delivered over 1 h; right). For control of the local effect in duodenum, see Extended Data Fig. 9. **e**, Quantification of preference at 1 h with PBS (pretest/posttest), vehicle and glutamate receptor inhibitors KA/AP3. KA/AP3 significantly reduced sucrose preference compared to pretest (*P* = 0.0294), posttest (*P* = 0.0497) and vehicle (*P* = 0.0294). **f**, Quantification of total intake during glutamatergic inhibition at 1 h. KA/AP3 significantly reduced sucralose intake (*P* = 0.0090). For **d**–**f**, *N* = 4 wild-type mice; *P < 0.05 by Kruskal–Wallis test with non-parametric comparisons using the Wilcoxon method. Shaded regions/error bars indicate s.e.m.

**Extended Data Fig. 6:**
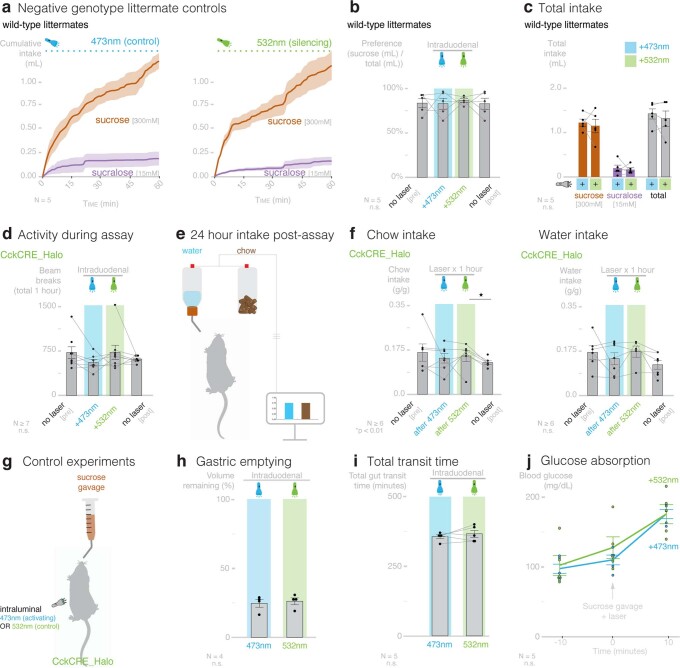
Laser inhibition of duodenal Cck-labeled neuropod cells does not cause malaise or off-target effects, related to Fig. 6. **a**, In CRE-negative littermate controls of CckCRE_Halo mice, average traces showing sucrose [300mM] and sucralose [15mM] consumption during control 473 nm light (left) and silencing 532 nm light (right). **b**, Preference quantified at one hour with no laser (pre/post), control 473 nm light, and silencing 532 nm light. **c**, Quantification of total intake at one hour (N = 5 littermate controls; n.s. by repeated measures ANOVA). **d**, Activity was measured as total beam breaks in x-y plane during one-hour choice assay with control 473 nm (N = 7) or silencing 532 nm light (N = 8; n.s. by repeated measures ANOVA). **e**, Water and chow intake were measured during the 23 hours following the choice assay. **f**, Total chow and water intake in 23 hours following the one-hour choice assay with no laser (pre, post), with control 473 nm light (N = 7), or with silencing 532 nm light (N = 6). Silencing 532 nm light did not affect subsequent intake of chow or water compared to control 473 nm light (CckCRE_Halo mice, *p = 0.0030 by repeated measures ANOVA with two-tailed paired t-test post-hoc analysis). **g-j**, CckCRE_Halo mice underwent gavage with 300 μL of sucrose [300mM] and were tested for off-target effects of laser inhibition. Laser inhibition with silencing 532 nm light, compared to control 473 nm light, did not affect (**h)** gastric emptying (N = 4), **(i)** total gut transit time (N = 5) or **(j)** glucose absorption (N = 5) after sucrose gavage (CckCRE_Halo mice, n.s.). Bold line = mean, shaded regions/error bars = S.E.M.

In the presence of 532-nm light, control littermates showed 90.8% (±3.7%) sucrose preference (*N* = 5 mice; not significant compared to controls; Extended Data Fig. 6a,b), whereas in CckCRE_Halo mice, sucrose preference was only 58.9% (±3.9%) (*N* = 8 mice; *P* < 0.01 compared to controls; Fig. 6a,b). In control experiments, silencing duodenal CCK-labeled neuropod cells with 532-nm light did not cause malaise, as neither locomotor activity during the assay (Extended Data Fig. 6d) nor chow or water intake in the following 24 h (Extended Data Fig. 6e,f) were affected. Additional experiments showed that laser inhibition with 532-nm light did not affect gastric emptying of sucrose, total gut transit time or glucose absorption compared to 473-nm control light (Extended Data Fig. 6g–j). Of importance, silencing CCK-labeled neuropod cells decreased sucrose intake and increased sucralose intake, but the total consumption of liquid during the 1-h test was not affected (*P* < 0.05; Fig. 6c and Extended Data Fig. 6c). In other words, silencing duodenal neuropod cells eliminated preference for sucrose over sucralose. In posttest controls without laser treatment, mice displayed the same pretest preference for sucrose (Fig. 6b), indicating that the animals did not lose their preference for sucrose but rather their ability to discern the preferred sugar from the sweetener.

**Extended Data Fig. 7:**
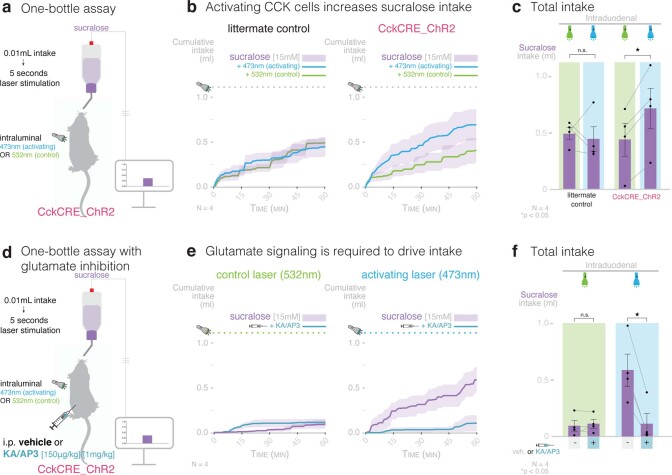
Glutamatergic signaling from Cck-labeled neuropod cells drives intake. **a**, CckCRE_Channelrhodopsin (CckCRE_ChR2) mice and littermate controls were given a one-bottle intake test of sucralose [15mM] for 1 hour with control 532 nm or activating 473 nm light. Laser stimulation was paired to solution consumption: for every 0.01 mL intake, mice received 5 seconds of intraluminal stimulation at 40Hz. **b**, In littermate controls (left) or CckCRE_Chr2 mice (right), average traces show intake of sucralose plus control 532 nm light or activating 473 nm light. **c**, Stimulation of duodenal Cck-labeled neuropod cells with 473 nm light increased intake of sucralose [15mM] (N = 4 mice; *p = 0.0062, repeated-measures ANOVA with post-hoc two-tailed paired t-test). **d**, In CckCRE_ChR2 mice, ionotropic/metabotropic glutamate receptor inhibitors KA/AP3 (150 μg/kg] /[1mg/kg] in 10 μL/g mouse in 1M NaOH in PBS, pH=7.4) or vehicle (1M NaOH in PBS, pH=7.4) were administered intraperitoneally 25 minutes prior to the one-bottle assay as in **(a)**. **e**, In CckCRE_ChR2 mice, average traces show intake of sucralose with vehicle (purple) or glutamate receptor inhibitor (blue) and control 532 nm light (left) or activating 473 nm light (right). **f**, Glutamate receptor inhibition reverses the increase in sucralose intake caused by optogenetic stimulation of Cck-labeled neuropod cells (N = 4 mice; *p = 0.0054, repeated-measures ANOVA with post-hoc two-tailed paired t-test). Bold line = mean, shaded regions/error bars = S.E.M.

To determine whether activating duodenal neuropod cells would increase an animal’s consumption of the non-preferred solution, sucralose, we bred mice in which CCK-labeled neuropod cells expressed channelrhodopsin 2 (CckCRE_ChR2). This excitatory opsin is activated by blue light (473 nm). The mice were presented with one bottle containing sucralose (15 mM), and intake of 0.01 ml triggered a 5-s laser stimulation (5 V, 40 Hz, 20% duty cycle) (Extended Data Fig. 7a). In this assay, 473 nm light had no effect on the intake of wild-type littermates (*N* = 4; not significant compared to 532-nm control; Extended Data Fig. 7b,c). However, in CckCRE_ChR2 mice, exciting CCK-labeled neuropod cells with 473-nm light significantly increased sucralose intake (*N* = 4; *P* < 0.05 compared to 532-nm control; Extended Data Fig. 7b,c). These results indicate that stimulating duodenal neuropod cells drives mice to consume sweetener as if it were sugar.

### The neurotransmitter for sugar preference is glutamate

**Extended Data Fig. 8:**
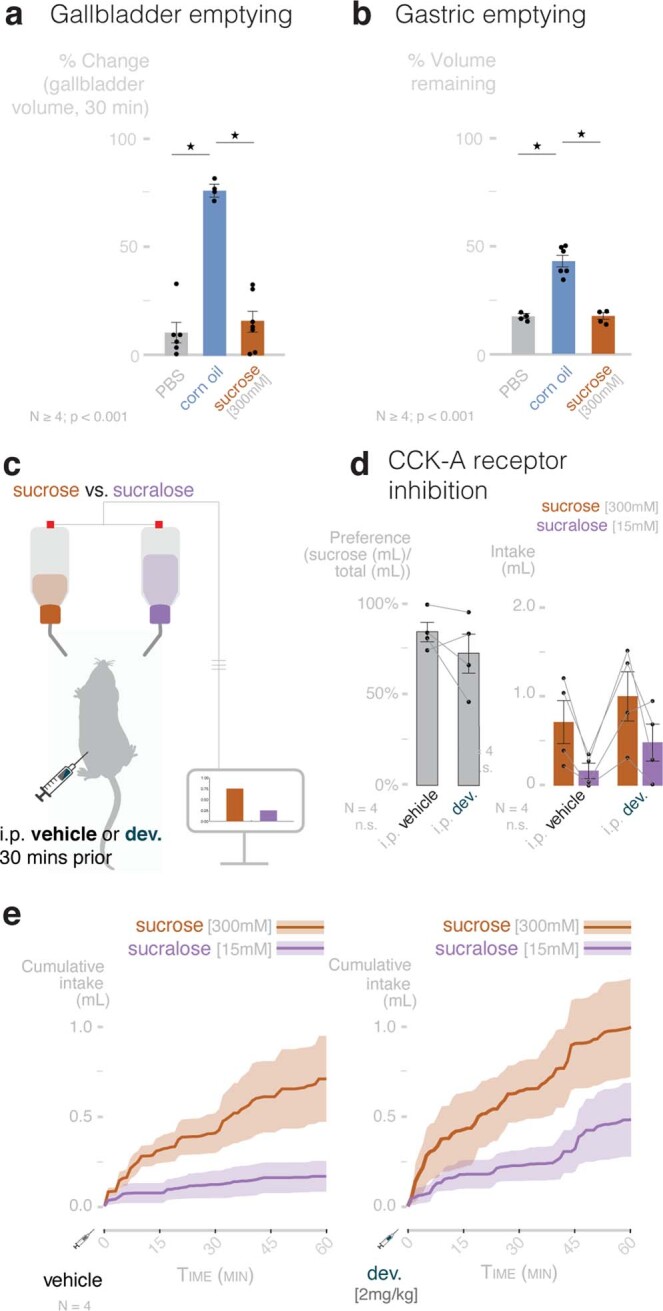
Cholecystokinin signaling does not mediate sucrose preference, related to Fig. 6. Circulating cholecystokinin is known to promote gallbladder emptying and slow gastric emptying in response to fat. **a**, Wild-type mice were anesthetized and PBS (negative control, N = 6), corn oil (positive control, N = 4), or sucrose ([300 mM], N = 7) was perfused into the duodenum and change in gallbladder volume was measured. Corn oil stimulated gallbladder emptying, while sucrose and PBS had no effect. p < 0.0001 by ANOVA. **b**, Wild-type mice were gavaged with 300 μL of PBS (negative control, N = 4), corn oil (positive control, N = 6), or sucrose ([300mM], N = 4) and gastric emptying was measured. Corn oil reduced gastric emptying (increased volume remaining), while sucrose and PBS had no effect. p < 0.0001 by ANOVA. **c**, Wild-type mice were given a two-bottle preference test between sucrose [300mM] and sucralose [15mM] for one hour. Cholecystokinin-A receptor inhibitor devazepide [2mg/kg] or vehicle (5% DMSO in PBS) was administered intraperitoneally (10 μL/g mouse) 30 minutes prior to assay. **d**, Preference for sucrose over sucralose (left) and sugar intake (right) during the one-hour assay with vehicle or devazepide. Preference was unchanged by devazepide compared to vehicle. Sucrose intake trended towards increasing with devazepide (N = 4 mice per group, n.s.). **e**, Average traces show sucrose and sucralose intake with intraperitoneal injection of vehicle (left) or cholecystokinin-A receptor inhibitor devazepide (right). Bold line = mean, shaded regions/error bars = S.E.M.

We next sought to identify the signaling molecules underlying preference for sugar over sweetener. The effect was not due to local or hormonal CCK signaling because sucrose perfusion through the small intestine did not alter physiological processes controlled by CCK, including gallbladder contraction (Extended Data Fig. 8a) and gastric emptying (Extended Data Fig. 8b). Indeed, blocking CCK-A receptors with intraperitoneal devazepide (2 mg kg^–1^) during the choice assay did not affect sucrose preference (*N* = 4 wild-type mice; not significant compared to vehicle controls; Extended Data Fig. 8c–e). This finding supports data from previous studies showing that CCK signaling does not contribute to conditioned sugar preference^30^.

We tested if glutamate signaling is required for CCK-labeled neuropod cells to drive sugar intake. Before gaining access to one bottle of sucralose, CckCRE_ChR2 mice were intraperitoneally injected with ionotropic and metabotropic glutamate receptor blockers (150 μg kg^–1^ KA/1 mg kg^–1^ AP3). The increase of sucralose intake driven by optogenetic excitation of CCK-labeled neuropod cells was blocked when glutamate receptors were inhibited (*N* = 4; *P* < 0.05; Extended Data Fig. 7d–f).

**Extended Data Fig. 9:**
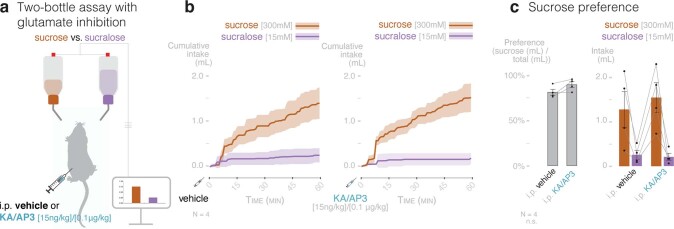
Duodenal local dose of glutamate receptor inhibitors does not impact sucrose preference when delivered systemically. **a**, Wild-type mice were given a two-bottle preference test between sucrose [300mM] and sucralose [15mM] for one hour. Local dose ionotropic/metabotropic glutamate receptor inhibitors KA/AP3 ([15ng/kg]/[0.1 μg/kg] in 10 μL/g mouse in 1M NaOH in PBS, pH=7.4) or vehicle (1M NaOH in PBS, pH=7.4) was administered intraperitoneally 10 minutes prior to assay. **b**, Average traces show sucrose and sucralose consumption with intraperitoneal injection of vehicle (left) or local dose glutamate receptor inhibitors KA/AP3 (right). **c**, Preference for sucrose over sucralose (left) and sugar intake (right) during the one-hour assay with vehicle or KA/AP3. Preference and intake was unchanged by systemic administration of local dose glutamate receptor inhibition compared to vehicle (N = 4 mice per group, n.s.). Bold line = mean, shaded regions/error bars = S.E.M.

To test the role of glutamate in preference for sugar over sweetener, a catheter was implanted into the duodenal lumen of wild-type mice to deliver a local dose of glutamate receptor blockers (15 ng KA and 0.1 µg AP3 in 0.4 ml; 10,000-fold lower than the dose used in Extended Data Fig. 7d–f). In mice receiving a vehicle control, sucrose preference was 82.4% (±3.2%). Inhibiting glutamate receptor signaling from the duodenal lumen significantly reduced sucrose preference to 44.0% (±5.2%) (*N* = 4; *P* < 0.05 compared to vehicle controls; Fig. 6d,e). Moreover, local inhibition of glutamatergic signaling also reduced sucrose intake and increased sucralose intake without significant changes in total intake (Fig. 6f). Control experiments showed that this local dose of glutamate receptor blockers did not affect sucrose preference when delivered systemically, confirming its local action in the gut lumen (Extended Data Fig. 9). Therefore, glutamatergic signaling from duodenal neuropod cells enables mice to discern sugar from sweetener.

Here, we demonstrate that an animal’s preference for sugar over sweetener depends on duodenal neuropod cells. These cells rapidly transduce such stimuli onto the vagus nerve using two receptors and two neurotransmitters; whereas sweetener activates T1R3 to cause the release of ATP, the entry of sugar into the cell stimulates the release of glutamate. By developing a flexible fiber for gut optogenetics, we discovered that sugar preference depends on neuropod cell glutamatergic signaling (see video https://youtu.be/q9KTD3TyAcs). Uncoupling the synapses between neuropod cells and vagal neurons will inform how appetitive functions beyond choice are continuously modulated by fast neurotransmission from the gut epithelium.

## Discussion

In his classic book *Behave*, the neuroendocrinologist Robert Sapolsky states ‘What occurred in the prior seconds to minutes that triggered the nervous system to produce the behavior, this is the world of sensory stimuli, much of it sensed unconsciously’^39^. While the sight, smell and taste of food change our perception of flavor^40^, such stimuli perceived in the minutes before consumption only partially explain our behavior^41^. In the case of sugar, the neurons in the brain driving our preference have received much attention. How we behave in front of sugars depends on a cascade of neuronal activity, including inputs from midbrain neurons that release reinforcing dopamine^2,42^, hypothalamic melanin-concentrating neurons^43^, brainstem neurons in the caudal nucleus tractus solitarius^33^ and vagal nodose neurons^33,44^. However, the identity of the cells in the gut that transduce the sensory stimuli to guide the animal’s choices have remained unknown.

Soon after sweet taste receptors were identified^23^, scientists sought to create sweet-blind mice by knocking out taste receptors only to discover that animals were still capable of discerning sugar^45^. Subsequent work has confirmed that oral sweet taste is not essential to drive sugar intake^1–3^. While duodenal infusions of sweetener and sugar activate separate hindbrain^33^ and striatal^46^ pathways, only sugar infusions drive a strong conditioned preference. The sugar effects are localized to the proximal small intestine because isolated sugar infusions into the ileum or restricted to the stomach do not condition a strong preference^10,47^. Recently, it was established that duodenal neuropod cells use the neurotransmitter glutamate to transduce d-glucose from gut to brain^14^. Their contribution to sugar preferences was unknown.

A major roadblock to study the real-time contribution of a specific gut sensory cell to behavior had been the lack of suitable tools. Unlike olfactory receptor neurons or taste receptor cells, neuropod cells are not clustered in one location. Instead, these cells are scattered throughout the intestinal epithelium. Identifying and manipulating them in freely moving animals is therefore difficult. Pharmacological tools alone are not specific to cell type given the ubiquitous expression of cell surface receptors such as SGLTs. A suitable tool to determine the contribution of specific neuronal cell to behavior is optogenetics. Bringing optogenetics to the gut lumen required the development of a new device. The flexible fiberoptic developed here allowed for the use of optogenetics to interrogate the contribution of gut sensations to behavior. We believe this device will allow scientists to determine how behavior is modulated by other visceral organs that are in constant motion, such as the heart, lung or bladder.

Aided by these technologies, we discovered that the sensory function of neuropod cells is akin to the role of taste receptor cells in detecting tastants so the animal can discern flavor or retinal cone cells in detecting light wavelength so the animal can discern color. Like other sensory transducers^11,23,48^, neuropod cells use different receptors and transmitters to sense and convey signals from specific stimuli. This work serves as a foundation to determine how other stimuli, such as fats, proteins or microbial molecules, are sensed and transduced in different regions of the intestine to drive appetitive decisions.

Together, the fast neurotransmission and slow endocrine actions of sensor cells provide a synergistic complement for the gut to influence the emotion and logic behind food choices. After all, despite how sweet a food may look, smell or taste, a gratifying experience requires a gut sensation.

## Methods

### Mouse strains

All experiments on mice were performed following approval by the Institutional Animal Care and Use Committee at Duke University Medical Center under protocol A280-18-12. Mice were group housed in Duke University’s Division of Laboratory Animal Resources, where they were kept on a 12-h light/12-h dark cycle (7:00–19:00) with access to water and standard mouse chow (Purina 5001) ad libitum, unless otherwise indicated. The facility maintained an ambient temperature of 18–23 °C and humidity of 40–60%. Male and female adult mice aged 6–20 weeks were used in all experiments. In behavioral assays, animals of similar age and sex were used in experimental and control groups. Mouse strains, source, background and stock number used to breed experimental mice are listed below. The following experimental mouse strains were purchased, received or bred in-house and used directly: C57BL6/J (Jackson Laboratories, stock number 000664), Swiss Webster (Charles River Laboratories, stock number 024) and CCK–GFP (courtesy of Dr. Rodger A. Liddle at Duke University, Swiss Webster background^49^). The following double-transgenic mouse strains were bred in-house: CckCRE_tdTomato, CckCRE_Halo-YFP, CckCRE_ChR2 and Neurod1CRE_Salsa6f. The following experimental mouse strains were purchased to breed the transgenic strains: CckCRE (Jackson Laboratories, C57BL/6J background, stock number 012706), Neurod1CRE (Jackson Laboratories, C57BL/6J background, stock number 028364), LSL_tdTomato (Jackson Laboratories, C57BL/6J background, stock number 007914), LSL_Halo-YFP (Jackson Laboratories, C57BL/6J background, stock number 014539), LSL_ChR2-tdTomato (Jackson Laboratories, C57BL/6J background, stock number 012567) and LSL_Salsa6f (Jackson Laboratories, C57BL/6J background, stock number 031968).

### Human samples

Human duodenal samples were obtained from the Duke University Medical Center Biorepository and Precision Pathology Center under the Institutional Review Board protocol Pro00035974 via anonymous tissue release. Per this protocol, informed consent was obtained from all study participants. All samples were deidentified, and all links to additional individual information were broken before receipt of fresh surgical specimens. Following surgical extraction, samples were placed in sterile PBS and stored at 4 °C before crypt dissociation.

### Vagus nerve recordings

Whole-nerve recordings were performed in wild-type mice (*N* = 5–11 per group), CckCRE_Halo-YFP mice (*N* = 5 per group) and CckCRE_Halo-YFP mice following fiberoptic implantation (*N* = 4–5 per group). Whole-nerve electrophysiology recordings of the cervical vagus nerve were performed as previously reported^14^. A 20-gauge gavage needle with two connected tubes for PBS perfusion and stimulant delivery was surgically inserted through the stomach wall into the duodenum or, for controls, the distal ileum (3 cm proximal to the cecum) or the proximal colon distal to the cecum. A perfusion exit incision was made at the ligament of Treitz for the small intestine or just proximal to the rectum for colon. PBS was constantly perfused through the isolated intestinal region at ~400 μl min^–1^ as a within-subject baseline and volume pressure control. Stimulation conditions were applied after recording 2 min of baseline activity. During nutrient stimulation conditions, PBS perfusion was continuous, and 200 μl of stimulant was perfused over 1 min using a syringe pump (Fusion 200, Chemyx). The 1-min infusions of each ligand were separated by at least 6 min or the return to baseline firing rate, whichever came first. Throughout experiments, sucrose response was used as a positive control. For all nutrient and laser stimulation conditions, data were excluded if a stable sucrose response was not seen throughout the recording session. Each mouse received one to four separate ligands in addition to the sucrose positive control. The order of subsequent ligands was random within mice. Each mouse received only one inhibitor. The preinhibitor and postinhibitor ligand infusions were within subject. All ligands were dissolved in PBS. The following final concentrations for each infused nutrient were used: 8% maltodextrin^6^, 150 mM α-MGP^6,33,50^, 15 mM sucralose^6,42^, 15 mM acesulfame K^33^, 30 mM saccharin^33^, 300 mM sucrose^42^, 150 mM d-glucose^6,42^, 150 mM d-galactose^6,33,50^ and 150 mM d-fructose^6,33,50^.

#### Data acquisition

Extracellular voltage was recorded as previously described^14^. The raw data were analyzed using SpikeTailor, a custom MATLAB software (MathWorks) script^14^. Spikes were detected using a threshold detected based on RMS noise. The firing rate was calculated using a Gaussian kernel smoothing algorithm in 200-ms bins^51^.

#### Optogenetic inhibition

A standard stiff silica fiberoptic cable (FT020, ThorLabs; power, 1.98 mW mm^–2^) was threaded through or along the gavage needle into the duodenal lumen. Laser stimulation was applied simultaneously with nutrient infusion. The laser was pulsed for 1 min at 40 Hz with a 5-V peak and 20% duty cycle (473-nm, 80-mW laser, RGBlase; 532-nm, 80-mW laser, RBGlase). Following recording of a preinhibition response to the selected ligands, 532-nm light was applied as above while the selected ligand was perfused.

#### SGLT1, T1R2/T1R3 and SGLT2 inhibition

For apical receptor inhibition, the SGLT1 competitive inhibitor phloridzin dihydrate (Sigma) or the sweet taste receptor inhibitor (T1R2/T1R3) gurmarin (Peptides International) were dissolved into 1 M sucrose, 45 mM sucralose or 450 mM α-MGP. Following recording of a preinhibitor response to the selected ligand, sucrose, sucralose or α-MGP were perfused with the apical receptor inhibitors for a final phloridzin concentration of 3 mM (ref. ^14^) and a final gurmarin concentration of 7 μΜ (refs. ^27,52^). SGLT2 inhibitor dapagliflozin (3 nM) in PBS was perfused through the lumen.

#### Neurotransmitter receptor inhibition

The following neurotransmitter/neuropeptide receptor blockers were used: CCK-A receptor antagonist devazepide (2 mg kg^–1^ in 5% DMSO PBS; Sigma)^14,53^, cocktail of the ionotropic glutamate receptor antagonist KA (150 μg kg^–1^ in PBS, stock made in 1 M NaOH then diluted, pH 7.4; Sigma)^14,54^ and the metabotropic glutamate receptor antagonist AP3 (1 mg kg^–1^ in PBS, stock made in 1 M NaOH diluted, pH 7.4; Sigma)^14,55^ and non-selective P2-purinoreceptor antagonist PPADs (25 mg kg^–1^ in PBS; Sigma)^32,56–58^. Following recording of a preinhibitor response, one inhibitor was delivered over 1 min (devazepide and PPADS were delivered at 10 μl g^–1^; the KA/AP3 cocktail was delivered at 20 μl g^–1^). Infusion of the selected sugar ligand was repeated for postinhibitor recording after an incubation period of 5–8 min for devazepide and 3–5 min for KA/AP3 and PPADs.

#### Data analysis

Stimulation response was quantified as the maximum firing rate after stimulation (stimulant conditions) or during recording (baseline). Time to peak was also quantified as the time from the start of infusion to the maximum firing rate for stimulant conditions, which evoked vagal firing. Each trial served as its own control by normalizing the firing rate to the prestimulus baseline firing rate (first 2 min of recording). Maximum firing rate, time to peak and area under the curve were analyzed across stimulation condition.

### Dissociation and isolation of single intestinal epithelial cells

Small intestines of mice were dissociated for single-cell RT–qPCR (CCK–GFP; *N* = 3 mice), calcium imaging (CckCRE_tdTomato; *N* = 8 mice) or in vitro electrophysiology (CckCRE_tdTomato; *N* = 9 mice) as previously described^14^. Briefly, the proximal half of the small intestine was removed, flushed with cold PBS and cut into sections. Tissue was shaken in 3 mM EDTA in PBS for 15 min at 4 °C followed by a 15-min incubation at 37 °C. The epithelium was then mechanically detached from the muscle by shaking in cold PBS. Following centrifugation at 800 r.p.m. (Eppendorf 5702 RH; rotor A-4-38), the pellet was resuspended and incubated in HBSS with dispase and collagenase for 10 min at 37 °C. Samples were then centrifuged (500 r.p.m.), filtered twice through 70-μm and 40-μm filters and resuspended in L15 medium (5% fetal bovine serum (FBS), 10 μl ml^–1^ 10 mM HEPES, 2,000 U ml^–1^ penicillin/streptomycin and 100 μl of 1,700 U ml^–1^ DNase) to produce a single-cell suspension for further analysis.

### CCK cell and vagal nodose neuron culture

The small intestines of CckCRE_tdTomato mice were dissociated to single cells as described in Dissociation and isolation of single intestinal epithelial cells. Cells were sorted using fluorescence activated cell sorting (BD FACSAria) selecting for tdTomato^+^ fluorescent cells. Cells were sorted into coculture medium (1× GlutaMAX, 10 mM HEPES, 200 U ml^–1^ penicillin/streptomycin, 1× N2 supplement, 1× B27 supplement, 10 ng ml^–1^ nerve growth factor (NGF), 25 ng ml^–1^ epithelial growth factor (EGF), 50 ng ml^–1^ noggin and 100 ng ml^–1^ R-spondin in Advanced DMEM/F-12). Sorted cells were plated on 2.5% Matrigel-coated (Corning, 356231) 12-mm coverslips at a concentration of ~5,000–10,000 enteroendocrine cells per coverslip. Nodose neurons were dissociated from C57BL/6J wild-type mice as described in Calcium imaging of dissociated cells. Neurons in medium were plated evenly on up to eight coverslips with or without enteroendocrine cells. Patch clamp electrophysiology was performed 2–3 d after plating.

### Patch clamp electrophysiology

Enteroendocrine cells and nodose neurons were cocultured as described in the coculture section. Neurons alone were also cultured as described above onto coverslips. Coverslips were placed in the recording chamber filled with extracellular solution containing 140 mM NaCl, 5 mM KCl, 2 mM CaCl_2_, 2 mM MgCl_2_ and 10 mM HEPES (pH 7.4, 300–305 mosM). CckCRE_tdTomato cells were identified by red fluorescence and neurons by their morphology and lack of fluorescence. Recordings were made using borosilicate glass pipettes pulled to ~3.5 MΩ resistance. For voltage clamp recordings, intracellular solution contained 140 mM CsF, 10 mM NaCl, 0.1 mM CaCl_2_, 2 mM MgCl_2_, 1.1 mM EGTA, 10 mM HEPES and 10 mM sucrose (pH 7.25, 295 mosmol). Neurons were held at −50 mV for 2 min after patching in voltage clamp mode to stabilize cells. Membrane time constant, cell capacitance and voltage threshold were determined using 200-ms steps from −70 mV to +20 mV in 10-mV increments. Stimuli were delivered using the SmartSquirt Microperfusion system (Automate Scientific). Then, the SmartSquirt nozzle was brought to within 100 µm of the paired enteroendocrine cell. While extracellular solution was perfusing through the chamber (~2 ml min^–1^), either 20 mM glucose or 2 mM sucralose was applied onto the cells via the SmartSquirt needle. Baseline neuronal activity was recorded in voltage clamp mode for 2 min before exposure to either stimulus in alternating order for 30–60 s, followed by a wash with extracellular solution. After exposure to stimuli, neurons were retested with voltage steps as described above to confirm the health of the cell. Each coculture pair that responded to at least one stimulus was exposed a second time to confirm activity. A voltage step protocol described above was run before and after each stimulus application to ensure neuron health. Neurons that did not respond to voltage step were not included in the analysis.

#### Data acquisition

Recordings were performed at room temperature using a MultiClamp 700B amplifier (Axon Instruments), digitized using a Digidata 1550A (Axon Instruments) interface and visualized in pClamp software (Axon Instruments). Data were filtered at 1 kHz and sampled at 10 kHz.

#### Data analysis

Cell capacitance was calculated as *C*_m_ = (*τ* *×* *I*_0_)/∆*E*, where *τ* is the time constant of the decaying current transient, ∆*E* is the voltage step and *I*_0_ is the current transient relative to prepulse potential (Platzer, 2016, 123). To account for cell variability and health, max current was normalized to the cell capacitance. Data are presented as the mean ± s.e.m. in log scale. Significance was determined using a two-tailed Student’s *t*-test with *α* = 0.05.

### Calcium imaging of dissociated cells

For neurons, C57BL/6J (*N* = 3 mice) nodose neurons were dissociated and plated as previously described^14^. Briefly, nodose ganglia were dissected and immediately placed into 500 μl of ganglia dissociation solution containing 10 mM HEPES, 1× GlutaMAX, 1× N2 supplement, 1× B27 supplement, 0.5 μg ml^–1^ NGF and 55 μg ml^–1^ Liberase (Roche, 5401054001) in Advanced DMEM/F-12. Following digestion, ganglia were rinsed twice with PBS, mechanically dissociated in dissociation solution and filtered through a 70-μm cell strainer. Cells were then plated on 12-mm coverslips and placed in a 37 °C incubator overnight. Cells were imaged 1–2 d after plating. For enteroendocrine cells, CckCRE_tdTomato (*N* = 8 mice) cells were dissociated as described in Dissociation and isolation of single intestinal epithelial cells and fluorescence sorted (BD FACSAria) selecting for tdTomato^+^ fluorescent cells. Cells were then plated on coverslips coated with 2.5% Matrigel (Corning, 356231). Enteroendocrine cells were imaged 2–6 h after plating. To load cells with calcium dye, cells were washed once with calcium-free PBS and incubated for 45 min at 37 °C with 5 μM Fluo-4 AM and 5 µM Fura Red AM calcium dyes (Life Technologies) and 0.1% Pluronic F-127 (Life Technologies) in imaging buffer (120 mM NaCl, 3 mM KCl, 2 mM CaCl_2_, 2 mM MgCl_2_, 10 mM HEPES, 10 mM glucose; 305 mosM ± 3 mosM). The loading buffer was then removed, and cells were washed twice with imaging buffer and placed in the dark for 15 min until they reached room temperature. Coverslips were placed in the recording chamber of a Zeiss Examiner Z1 and imaged with a Hamamatsu camera (Orca-flash4.0, C11440) using the Zeiss ZEN Blue software package. Fluo-4 and Fura Red emission images were obtained using 480-nm and 570-nm excitation, respectively. Images were collected at 1.5-s intervals with a 100-ms exposure time. Each recording was 210 s (3.5 min) long. Imaging buffer was continuously perfused (~2 ml min^–1^) over the coverslips throughout the imaging session. Two stimuli were applied during each recording, 20 mM d-glucose and 2 mM sucralose. Stimuli were each delivered for 15 s with 30 s of buffer perfused in between each stimulus. The order of the experimental stimuli was alternated to offset potential order effects. Each recording session concluded with 50 mM KCl as an activity control (KCl concentration was achieved by substituting for NaCl and not an addition of more KCl). A response to KCl was defined as a ratio increase >10% above baseline. Cells that did not reach this KCl threshold were not included in the analyses.

#### Analysis

Fluorescence values for each individual cell were calculated as the mean fluorescence intensity in a user-defined region of interest in Fiji software. Intracellular calcium changes were then calculated as ∆*F*/*R* (∆*F* = change in fluorescence of the ratio *R*; *R* = Fluo-4/Fura Red) based on baseline fluorescence. Ratiometric values were then normalized to the peak KCl response. A positive response was defined as an increase in ratio >10% above baseline. If the cell responded to a second stimulus, a new baseline of five frames before application was calculated, and a response was calculated as >10% from the new baseline. A Student’s paired *t*-test was used for a single comparison between stimuli and KCl. Significance was set at *P* < 0.05. Statistical analyses were performed using MATLAB software (MathWorks), and graphs were made with Microsoft Excel.

### In vivo calcium imaging of vagal nodose neurons

In vivo vagal nodose imaging was performed in Neurod1Cre_Salsa6f (*N* = 4) mice where GCaMP6f is expressed in vagal neurons. Nodose imaging was performed as previously described^17^. Mice were initially anesthetized with urethane (2 mg g^–1^) and maintained with 0–0.5% isoflurane as needed throughout the procedure. The right nodose ganglion was partially excised and placed on a metal platform, stabilized with silicone elastomer (Kwik-Sil) and covered with a 5-mm coverslip. A 20-gauge gavage needle attached to a gravity perfusion system was surgically inserted through the stomach wall into the duodenum. A perfusion exit incision was made at the ligament of Treitz. PBS was constantly perfused at <1 ml min^–1^ for the duration of the recording as a within-subject baseline and volume pressure control. Calcium transients were imaged using a multiphoton microscope (Bruker Ultima IV) using a ×16 objective. Laser wavelength was set to 920 nm, and frames were captured at a rate of 683 ms per frame. Baseline activity was imaged while perfusing PBS. Sucrose (300 mM) and sucralose (15 mM) stimuli were perfused back to back and in reverse order to determine whether the same cell or different cells responded to both sugars. In addition, mannitol (300 mM) was used as an osmolarity control. Stimuli were delivered for 60 s with 2 min of baseline before and after application.

#### Data analysis

Fluorescence values for each individual neuron were calculated as the mean fluorescence intensity in a user-defined region of interest using Fiji software. Intracellular calcium responses were calculated as ∆*F*/*F* = (*F* *–* average *F* of entire run)/average *F* of entire run. Stimulation response was quantified as the maximum ∆*F*/*F* after stimulation onset. A positive response was defined as an increase of >20% over baseline within each neuron.

### Single-cell RNA sequencing

Left (*N* = 6) and right (*N* = 5) nodose ganglia from adult C57BL/6J wild-type euthanized mice were dissected as described in Calcium imaging of dissociated cells and separated into two distinct tubes. Dissections were completed in tandem by three lab members, and all nodose ganglia were dissected within 30 min, at which point 55 μg of Liberase (Roche) was added to each tube. Ganglia were dissociated into single cells as described in the coculture section. The dissociated solution was then carefully laid on a density gradient of 500 μl of 12% and 500 μl of 28% Percoll (Sigma) and centrifuged for 10 min at 2,900*g* at room temperature. Once centrifugation was complete, the top 700 μl was removed, and 700 μl of fresh dissociation solution was added. Cells were then centrifuged for 15 min at 2,900*g*, and the final pellet was resuspended in 500 μl of PBS + 0.04% bovine serum albumin (BSA) and passed to the Duke University Human Vaccine Institute Sequencing Core for further processing. Capturing of single cells was performed using Chromium Single-Cell 3′ v2. cDNA synthesis with PCR and library preparation were done according to the manufacturer’s guidelines. Libraries were sequenced on an Illumina NextSeq 500. The Cell Ranger pipeline version 2.1.1 was used with the mm10 mouse reference genome version 2.1.0 to convert base calls to fastq format and align, map and count genes.

#### Analysis of nodose single-cell sequencing data

RStudio package Seurat version 3.1.0 was used for the analysis^59^. We integrated our data set with the published atlas for the nodose ganglia as a reference^60^. A total of 5,847 cells were sequenced with a mean of 137,352 reads and 2,817 genes detected per cell. Cells were filtered for gene content (fewer than 1,000 or greater than 6,000 genes detected were removed) and mitochondrial content (greater than 10%), leading to the removal of 340 cells before merging the left and right nodose data sets^59^. Normalization, feature selection, scaling and linear dimensional reduction were then performed using default parameters in Seurat. Transfer ‘anchors’ between our data set and the reference published set were determined using the algorithm implemented in Seurat, and we classified our cells into the 24 clusters previously identified. Cluster identities were confirmed with expression levels of cluster markers, and gene expression levels across clusters were visualized using uniform manifold approximation and projection (UMAP). For glutamate and ATP receptor genes, we determined composite gene expression of ionotropic (*Gria*, *Grik*, *Grin* and *P2rx*) and metabotropic (*Grm* and *P2ry*) family receptor genes by summing counts for the respective genes in each cell. All reported expression profiles were confirmed in both data sets as well as the pooled data set. Only sequencing data generated for this publication are presented in the figures.

### Single-cell RT–qPCR

RNA isolation from single cells was performed using the Cells Direct One-Step qRT–PCR kit (CDK kit, Thermo Fisher) per the manufacturer’s protocol. Lysis Buffer Mix (5 μl) was pipetted into each well of a 96-well plate and centrifuged at 500*g* to spread buffer. Following the dissociation protocol, single cells were sorted into a U-bottom 96-well plate (Sigma) based on GFP signal using a MoFlo XDP sorter. For each mouse, 60 GFP^+^ cells, 30 GFP^–^ cells and control wells were sorted. Control wells of 0, 10 and 100 cells were run in duplicate. Following sorting, the contents of each well were pipetted into a 96-tube, 0.2-ml PCR plate, which was then incubated in a thermocycler at 75 °C for 10 min. After centrifuging to pellet, DNase I and 10× DNase I reaction buffer from the CDK kit were added to each well and incubated at 25 °C for 5 min. Two microliters of 25 mM EDTA was added to each well, vortexed and pelleted. The plate was then incubated at 75 °C for 10 min to inactivate DNase I. Next, cDNA was synthesized and preamplified. Specific Target Amplification (STA) mix was made by mixing 1 μl of each TaqMan probe. STA mix, superscript reverse transcriptase (RT) and reaction buffer from the CDK kit were added to each sample and incubated on a thermocycler for 15 min at 50 °C, 2 min at 95 °C and 20 cycles of 15 s at 95 °C and 4 min at 60 °C. Gene expression was then probed using the 96.96 Dynamic Array integrated fluidic circuit on a Biomark using the manufacturer’s protocol (Fluidigm).

#### Quality control

Quality of the threshold cycle (*C*_t_) values from the Biomark output was assessed using the Fluidigm Real-Time PCR Analysis software (Fluidigm). All trials (*N* = 3 mice; *n* = 60 positive cells and 30 negative cells per mouse) were loaded simultaneously for analysis. The quality was analyzed in linear derivative mode, and the quality threshold was set at 0.65 based on the manufacturer’s recommendations. All curves not meeting the quality threshold were analyzed visually for smoothness (more smooth representing high quality) and entered into analysis based on comparison with passing curves. All cells not meeting quality measures or having no detected transcripts for either housekeeping gene (*Gapdh* or *Actb1*) were excluded from analysis (48 positive cells and 24 negative cells were excluded).

#### Analysis

Further processing of *C*_t_ values was performed based on Stählberg et al.^61^. Relative quantities (RQ) of cDNA molecules were calculated using the formula RQ = 2(^*C*^_*q* cutoff_ – *C*_*q*_) using a *C*_*q*cutoff_ value of 34. The RQ value for any sample expressing no detectable transcripts for a gene was set at 0.5. All data were expressed in a log_2_ scale. Heat maps were generated for gene expression normalized within each gene (mean of 0; s.d. of 1) using Qlucore Omics Explorer (Qlucore). Differential gene expression analysis ((1) CCK–GFP^+^ versus CCK–GFP^–^, (2) *Slc5a1*^+^ versus *Slc5a1*^–^ in CCK–GFP^+^ cells and (3) *Tas1r3*^+^ versus *Tas1r3*^*–*^ from CCK–GFP^+^ cells) was performed using two-tailed two-group *t*-test comparisons with a *q* value cutoff of 0.05 (as implemented by Qlucore).

### Immunohistochemistry

CCK–GFP (*N* = 3) mice were transcardially perfused with PBS for 3 min followed by 4% paraformaldehyde (PFA) for 3 min at a rate of 600 µl min^–1^. Each small intestine was collected, opened lengthwise, rolled with the proximal end in the center and postfixed in 4% PFA for 3 h. Tissue was then dehydrated in 10% sucrose for 1 h and 30% sucrose for at least 12 h. Samples were embedded in optimal cutting temperature compound (OCT) (VWR) and stored at −80 °C. Tissue was sectioned onto slides at 14 μm using a cryostat. Tissue slides were postfixed in 10% normal buffered formalin (VWR) for 10 min and washed in Tris-buffered saline with 0.05% Tween-20 (TBST) (Sigma). SGLT1 staining was achieved by performing heat-mediated antigen retrieval. Trisodium citrate dihydrate buffer (10 mM in PBS, 0.05% Tween, pH 6.0; Sigma) was heated in a slide holder in a water bath to >90 °C. Tissue slides were immersed for 20 min and then immediately placed into cool tap water and washed in TBST for 5 min. Tissue was blocked in 10% donkey serum (Jackson ImmunoResearch) for 1 h. Tissue was then incubated with primary antibody dissolved in antibody dilution solution (PBS with 1% BSA and 0.0025% Triton-X 100) for 24 h at 4 °C and then 1 h at room temperature. The following primary antibodies and dilutions were used: anti-SGLT1 (rabbit, Abcam, ab14686; 1:100) and anti-GFP (chicken, Abcam, ab13970; 1:500). After primary antibody incubation, tissue was washed in TBST and incubated with secondary antibody in antibody dilution solution (1:250) for 1 h at room temperature. The following secondary antibodies were used: Alexa Fluor 488 AffiniPure F(ab′) Fragment Donkey Anti-Rabbit IgG (H+L) (Jackson ImmunoResearch, 711-546-152, RRID AB_2340619), Cy3 AffiniPure F(ab′) Fragment Donkey Anti-Rabbit IgG (H+L) (Jackson ImmunoResearch, 711-166-152, RRID AB_2313568) and Alexa Fluor 488 AffiniPure F(ab′) Fragment Donkey Anti-Chicken IgG (H+L) (Jackson ImmunoResearch, 703-546-155, RRID AB_2340376). Tissue was then washed with TBST, stained with DAPI (1:4,000) for 3 min, washed in TBST and mounted using Fluoro-Gel with Tris Buffer (Electron Microscopy Sciences). Imaging was done on a Zeiss 880 Airyscan inverted confocal microscope. Images were adjusted for brightness/contrast using ImageJ (Fiji).

### In situ hybridization

CCK–GFP (*N* = 3) mice were transcardially perfused with PBS for 3 min followed by 4% PFA for 3 min at a rate of 600 µl min^–1^. Each small intestine was collected, opened lengthwise, rolled with the proximal end in the center and postfixed in 4% PFA for 24 h. Tissue was then dehydrated in 10% sucrose and 30% sucrose for 24 h each. Samples were embedded in OCT (VWR) and stored at −80 °C. Tissue was sectioned onto slides at 14 μm using a cryostat. RNA detection was performed using the RNAscope Multiplex Fluorescent Reagent kit v2 Assay (ACD). Briefly, tissue slides were baked for 30 min at 60 °C and postfixed in 10% normal buffered formalin (VWR) for 60 min before being washed in PBS twice (Sigma). Slides were then dehydrated using successive ethanol washes (50%, 70%, 100% and 100%) for 5 min each. Slides were then incubated with hydrogen peroxide for 10 min before undergoing target retrieval using RNAScope reagents in a steamer. Slides were submerged into the RNAScope target retrieval solution at >99 °C for 5 min. Slides were then treated with protease III for 30 min at 40 °C before subsequent hybridization and amplification steps per the manufacturer’s instructions. The following probes used were all purchased from ACD: Mm-Cck (402278), Mm-Slc5a1 (468888) and Mm-Tas1r3 (515431). Hybridization signal was detected using Opal dyes (Akoya Biosciences) at a dilution of 1:1,500. Tissue was then washed with TBST, stained with DAPI (1:4,000) for 3 min, washed in TBST and mounted using Fluoro-Gel with Tris Buffer (Electron Microscopy Sciences). Imaging was done on a Zeiss 880 Airyscan inverted confocal microscope. Images were adjusted for brightness/contrast using ImageJ (Fiji). Cells with greater than two puncta within the cell body were considered positive for the gene. Control slides using the negative control probes (ACD) were used to ensure that background staining was less than one puncta per cell.

### Organoid culture

#### Murine organoids

Mouse organoids were cultured from CCK–GFP mice (*N* = 3) per Sato et al.^28^. Briefly, the proximal one-third of the small intestine was flushed with cold PBS, opened lengthwise and cut into ~1-cm pieces. Tissue pieces were incubated with 1.5 mM EDTA on ice for 25 min and then 37 °C for 15 min. Crypts were detached by shaking in cold PBS, pelleted at 100*g* and resuspended in Matrigel (Corning, 356231). Crypts were plated in 50-µl mounds in 24-well plates and maintained in organoid culture medium containing 1× GlutaMAX (Gibco), 10 mM HEPES (Gibco), 200 U ml^–1^ penicillin/streptomycin (Gibco), 1× N2 supplement (Gibco), 1× B27 supplement (Gibco), 1 mM *N*-acetylcysteine (Sigma), 50 ng ml^–1^ EGF (Peprotech), 100 ng ml^–1^ noggin (Peprotech) and 10% R-spondin conditioned medium (produced in-house using Trevigen 3710-001-K cells) in Advanced DMEM/F-12 (Gibco). Y-27632 (Enzo; 10 μM) was added to the culture medium for initial plating.

#### Human organoids

Human organoids were cultured per Fujii et al.^29^. Human tissue was washed in PBS, and the epithelial layer was dissected from submucosa and connective tissue and minced. All tips and tubes used were coated with FBS. Tissue pieces were washed in PBS until clear and incubated in 5 mM EDTA in PBS for 1 h on ice. Crypts were detached by shaking in cold PBS, pelleted at 100*g* and resuspended in Matrigel (Corning, 356231). Crypts were plated in 50-µl mounds in 24-well plates and maintained in Human Intesticult (Stem Cell, 06010) with 10 μM Y-27632 at initial plating.

### Glutamate release assay

Murine or human small intestinal organoids were passaged and plated into a 96-well plate in 25-μl Matrigel mounds in organoid culture medium as above. The human organoid medium contained 500 ng ml^–1^ human R-spondin (Peprotech) and was supplemented with 500 nM A-83-01 (Tocris) and 10 nM leu-gastrin (Sigma) for differentiation with 10 μM Y-27632 at passage^62^. When mature morphology was achieved 3–7 d after passage, medium was removed, and organoids were washed in PBS twice for 5 min at room temperature. Organoids were then stimulated with 60 μl of 300 mM sucrose, 150 mM α-MGP, 15 mM sucralose or PBS for 10 min at 37 °C. Supernatant was collected, centrifuged for 10 min at 13,000*g* to remove insoluble material and stored at −20 °C for up to 2 weeks. Glutamate concentration in samples was assessed using the Glutamate Release Assay kit (Sigma). Fifty microliters of each sample was mixed with buffer, glutamate enzyme mix and developer following the manufacturer’s protocol. Each experimental condition was run in triplicate on every plate. A glutamate standard was run for every plate. Control wells of sample and developer without enzyme mix were run in duplicate and included for each sample. Absorbance at 450 nm was measured on a plate reader (Tecan Infinite 200 Pro). Nine reads were taken per well and averaged. The absorbance from the control was subtracted from each experimental sample absorbance for the corrected value. Glutamate amount and concentration was calculated using the standard curve.

### Flexible fiberoptic implant fabrication

#### Waveguide fabrication

The step index core/cladding flexible polymer waveguide was fabricated using the thermal drawing process starting from a macroscopic polymer preform (template)^37,38,63^. The preform was assembled by inserting a PC rod (1/8" (3.81 cm) in diameter, McMaster-Carr) into a PMMA tube (1/4" (7.62-cm) outer diameter and 1/8" (3.81-cm) inner diameter; US Plastics) and then consolidating the rod-in-tube assembly at 170 °C in a vacuum oven. The resulting preform was drawn into a meters-long fiber in a custom-built fiber drawing tower at a temperature of 270 °C. The lateral dimensions of the preform were reduced by 30 times to produce a microscopic (220- to 230-μm-diameter) PC/PMMA core/cladding optical waveguide.

#### Physical characterization of flexible polymer waveguide

Optical transmission loss of the fibers was quantified by coupling the fibers to a diode-pumped solid state laser (Laserglow; 50-mW maximum output, wavelength *λ* = 473 nm) via ferrules, and the light output was measured by a photodetector (S121C, 400–1,100 nm, 500 mW; Thorlabs) attached to a power meter (PM100D, Thorlabs). Optical transmission was quantified for a range of fiber lengths (1–10 cm), bending angles (0°, 90°, 180° and 270°) and radii of curvature (0.5, 1, 2.5, 5, 7.5, 10, 12.5 and 15 mm).

#### Gut implant fabrication

To optically couple as-drawn fibers to a light source, 9- to 10-cm-long fibers were inserted into a 10.5-mm-long, 2.5-mm-diameter, 231-μm bore size ceramic ferrule (Thorlabs) and affixed with optical epoxy (Thorlabs). The ferrule edge was then polished using a Thorlabs fiber polishing kit. Fiber was then threaded through ~7.5 cm of microrenathane tubing (BrainTree Scientific) to provide structural stability for tunneling. The proximal ~6.75 cm of the tubing closest to the ferrule was opacified with liquid electrical tape (Starbrite) to reduce non-specific activation of CCK-expressing cells in the skin (Fig. 5h). The final length of the device was ~9.25 cm including the ferrule; ~1.5 cm of the device could be illuminated and ~0.75 cm of fiber extended beyond the tubing. The average power recorded from the device tip was measured using a photodetector (S140C, 250–1100 nm, 500 mW; Thorlabs) attached to a power meter (PM100D, Thorlabs). Average power output (optical intensity) at the end of the PC/PMMA fiber with a 5-V, 40-Hz, 532-nm laser input was 1.07 mW mm^–2^ before implantation.

### Gut fiberoptic implantation surgery

Adult CckCRE_Halo-YFP, CckCRE_ChR2 mice or littermates were singly housed and acclimated to behavioral cages (TSE PhenoMaster) 1 week before surgery. Mice were anesthetized with isoflurane (1–3% in oxygen). A 2-cm incision was made from the xiphoid process diagonally to the left-mid clavicular line. The peritoneal cavity was accessed, and the stomach was extracorporealized for implantation. A purse string suture was made in the gastric antrum, avoiding blood vessels. A small incision was made in the stomach within the suture, and a gavage needle was used to dilate the pylorus. The distal end of the device was threaded into the proximal duodenum so that the illuminated region of the device was in the proximal small intestine (Fig. 5h). The purse string stitch was tied to secure the device in the intestine. The opacified portion of the device was tunneled to the base of the skull. The peritoneum and overlying skin were sutured. The device exited the tunnel at the base of the skull and was skull mounted; skull mounting was required to maximize longevity of the implant. For maximal adhesion, the skull was etched with a razor blade, and a thin layer of Metabond cement (Clear L-Powder S399+catalyst; Metabond) was applied. Then, the Metabond layer was etched and the device attached using standard dental cement (Stoelting, 51458). Mice recovered for at least 5 d or until normal feeding behavior and activity returned.

#### Fiber durability

For all experiments using the flexible fiberoptic device, a total of 40 mice were implanted for experimentation. Of the 40 mice implanted, 36 had devices that were intact and functional (90% success rate). Failure was due to a break in the tip of the fiber where it exits the clear tubing (four total devices). Data from mice with broken fiberoptic devices were excluded from analyses.

### Duodenal catheter surgery

Adult C57BL/6J wild-type mice were surgically implanted with catheters into the duodenum, using a similar procedure as previously described^9,64^. Mice were anesthetized with isoflurane (1–3% in oxygen). A 2-cm incision was made from the xiphoid process diagonally to the left-mid clavicular line. The peritoneal cavity was accessed and the stomach extracorporealized for catheter implantation. Microrenathane tubing (BrainTree Scientific) with one silicone ball (Home Depot) at implantation end was inserted into a small incision made in the stomach within a purse string suture. The distal end of the catheter was threaded into the proximal duodenum, and the silicone ball was sutured inside the stomach to keep the catheter in place. The other end of the catheter was tunneled to the back and directed out in the small intrascapular incision. This end was secured in place with surgical mesh. The proximal end of the catheter was sealed with a metal cap. Mice were then singly housed and recovered for at least 5 d until normal feeding behavior and activity returned.

### Phenotyping equipment

All optogenetic behavior experiments were performed in a principal investigator-managed husbandry system. Animals were housed in a custom-built PhenoMaster behavioral phenotyping system (TSE Systems). The PhenoMaster was programmed (software version 6.6.9) to automatically maintain a light cycle (7:00 lights on; 19:00 lights off), temperature control (22 °C) and humidity control (40%). The PhenoMaster holds 12 clear cages in which animals were singly housed. Cages were industrially washed, and bedding (ALPHA-dri) was replaced weekly. Animals were provided with standard mouse chow (Purina 5001) and reverse osmosis water ad libitum unless fasted for a choice assay. All cages housed an enrichment device, which also served to weigh the animals. A food hopper, water bottle and weigh container were attached to weight sensors (TSE). Food intake, water intake and weight were automatically measured every 5 s to the nearest 0.01 g. For drinking measurements, a 10-s smoothing interval with a maximum raw analog-to-digital conversion counts difference of 40,000 was permitted. For weight measurements, a 15-s smoothing interval with a 15-g threshold and a maximum raw analog-to-digital conversion counts difference of 1,000,000 was permitted. Intake was measured every 5 s and binned every 1 min for analyses unless otherwise indicated. Animal activity was determined by beams crossed in the *x* and *y* planes and was collected with a 100-Hz scan rate. For optogenetic stimulation experiments, custom PhenoMaster software drove scheduled TTL pulses, which triggered laser on/off. For optogenetic experiments, TTL signals were set to be triggered every 3 min. Each cycle included 1 min on with a 40-Hz, 5-V pulse at 20% duty cycle and 2 min off. Each experimental session with laser stimulation began with 1 min of laser on. Following experiments, raw data were downloaded from the Phenomaster software and analyzed using MATLAB software (MathWorks). Unless otherwise indicated, all activity, food intake and water intake measurements were binned in 1-min intervals for analysis. Data were corrected for minor fluctuations by only permitting a monotonically increasing function for both food and water intake; values that represented negative food intake were replaced by the most recent value.

#### Plexiglass cage manufacturing

The choice assay paired with intraduodenal drug delivery occurred in in-house-manufactured clear plexiglass cages. Clear plexiglass (Home Depot, model acr0802448; 0.2 × 60.96 × 121.92 cm) was manufactured into cages with a 20.32 × 20.32-cm base and four 25.4-cm-tall walls secured with clear silicone (Loctite waterproof sealant). The walls were snap cut by hand in our laboratory or in the Duke Engineering machine shop. The top of the cage was open. To allow mice to move around the cage freely, a custom swivel arm (TSE), which introduced the tubing to attach to each catheter, was secured on one wall with a custom three-dimensional-printed device. This device was essentially a tube that snuggly fit around the metal swivel arm to hold it upright and was super-glued to one side of the cage.

### Food intake following lipid gavage

Adult CckCRE_Halo mice (*N* = 3) were implanted with the flexible fiberoptic and housed in the PhenoMaster. Following recovery, mice were habituated to gavage, handling and connection to the fiber patch cable. On experimental days, mice were gavaged with a fat solution (7% Intralipid diluted in PBS; Sigma) or PBS (delivered as 0.1 ml per 10 g) after 90 min of food and water restriction. Immediately following gavage, mice received inhibiting (532-nm) or control (473-nm) light stimulation (40 Hz, 5 V, 20% duty cycle). Thirty minutes after gavage, food and water was available for 3 h, during which the light stimulation continued. Food intake was continuously measured for 3 h. Food intake was calculated as gram of chow intake per gram body weight.

### Oral glucose tolerance test

Blood glucose was measured in adult CckCRE_Halo mice (*N* = 4) by an oral glucose tolerance test. Mice were food and water deprived for 1 h. Then, mice were gavaged with sucrose (1 M, as 0.1 ml g^–1^ mouse; approximate sucrose concentration consumed in 1-h choice assay) and received 10 min of 532-nm or 473-nm light stimulation (40 Hz, 5 V, 20% duty cycle). Blood glucose was measured (True Metrix 60 Blood Glucose Meter) after 1 h of deprivation (−10 min), immediately following gavage (0 min) and after 10 min of laser inhibition (10 min).

### Total gut transit time

Total gut transit time was performed as previously described^65^. Adult CckCRE_Halo mice (*N* = 4) were implanted with the flexible fiberoptic. Following recovery, mice were habituated to gavage, handling and connection to the fiber patch cable. On test days, mice were food and water restricted for 1 h. Mice then received a gavage of a solution containing Evans Blue (5%; Sigma) and methylcellulose (0.5%; Sigma) mixed in 300 mM sucrose in 1× PBS, followed by 1 h of silencing (532-nm) or control (473-nm) laser stimulation (40 Hz, 5 V, 20% duty cycle). Bedding was evaluated for a blue fecal pellet every 10 min. Total gut transit time was calculated as the time between the gavage and the first blue fecal pellet. At euthanasia, one device was broken, and data from that mouse were excluded from analysis.

### Gallbladder emptying

Gallbladder emptying, an effect of CCK, was measured by calculating gallbladder volume before and after stimulus perfusion to the duodenum. Serum levels of CCK were not measured due to the unreliability of commercial kits. Gallbladder volume was calculated using previously published reports^66,67^. Wild-type mice were fasted overnight before being fully anesthetized under isoflurane. A laparotomy was performed, and the gallbladder was gently exposed and measured using a microcaliper. Gallbladder volume was calculated using the formula gallbladder volume (μl) = length (mm) × width (mm) × depth (mm) × π/6 (ref. ^67^). A gavage needle was inserted and secured in the duodenum through the pylorus and was connected to an infusion pump (TSE Systems). Mice received an infusion of PBS (negative control), corn oil (positive control; Canola) or sucrose (300 mM) at 40 μl min^–1^ for 5 min. Thirty minutes later, gallbladder volume was measured again. Gallbladder emptying was calculated as the percent change from the pre- to postinfusion volume.

### Gastric emptying

Gastric emptying was measured as previously reported^68,69^. Wild-type mice were fasted overnight before receiving a 0.3-ml gavage of PBS (control), corn oil (positive control; Canola) or sucrose (300 mM). Fifteen minutes later, mice were killed, and the duodenum and esophagus were clamped and tied off securely with suture thread. The stomach was then removed and weighed. The stomachs were desiccated at 65 °C for 6 d before being weighed again. Gastric emptying was calculated as gastric emptying (% volume remaining) = (postweight/preweight) × 100. A separate cohort of CckCRE_Halo mice were implanted with flexible fiberoptic devices in the duodenum. Mice were food and water deprived for 1 h. They were then gavaged with PBS or sucrose (300 mM) and received laser stimulation (40 Hz, 5 V, 20% duty cycle) for 15 min, and stomachs were dissected as described above.

### Choice assay

Mice were given free access to 300 mM sucrose and 15 mM sucralose for 24 h in the home cage to control for neophobia. During 24-h access, mice had ad libitum access to food and water, although water intake was negligible; implanted mice were not connected to patch cables. For each subsequent choice assay, at the start of the dark cycle (19:00), mice were placed in a cage with fresh bedding and restricted of food and water either in the Phenomaster or plexiglass cages. One hour after onset of the dark cycle (20:00), 300 mM sucrose and 15 mM sucralose became available for free consumption for 1 h. Concentrations were selected based on prior studies showing iso-sweetness^6,42^. The side of the sucrose and sucralose solutions was swapped each test to control for side preferences. To advance to optogenetic or pharmacologic inhibition, mice were required to display a stable preference for sucrose, defined as >66% sucrose preference in two consecutive tests and not varying by more than 15% across both tests. For five mice who did not display a clear preference by the seventh test or displayed a clear side preference by the third test were reexposed for 24 h as above. The average number of test days to advance to inhibition was 5.24 d. Following each test session, mice were disconnected when appropriate (optogenetic and intraduodenal infusion tests) and given ad libitum access to food and water. The start of all test sessions was separated by at least 48 h.

#### Optogenetic inhibition

Implanted CckCRE_Halo-YFP mice (final *N* = 8, *N* = 5 male/3 female) and their wild-type littermates (final *N* = 5, *N* = 3 male/2 female) underwent baseline choice assays as described in Choice assay. Testing occurred in our TSE PhenoMaster apparatus. During all baseline and experimental assays, mice were connected to the laser using a custom swivel arm (TSE) coupled to a rotary joint patch cable (ThorLabs, RJPFF2) for free movement at the time of dark onset for consistency and acclimation. Once a stable preference was established, each mouse underwent two experimental conditions, 532-nm (silencing) light and 473-nm (control) light, followed by a repeated baseline. Order of wavelength was counterbalanced to control for order effect. During photostimulation conditions, the Phenomaster delivered a TTL pulse for laser stimulation: 1 min on (5 V, 40 Hz, 20% duty cycle) and 2 min off for the full hour. The following analyses were assessed: minute-to-minute sucrose and sucralose consumption, preference for sucrose relative to the total amount of volume consumed, motor activity and food/chow intake over 24 h following the assay. Fiberoptic placement and power output was confirmed at the end of the study. Only mice who completed all four tests and whose device had appropriate power/placement were included in analysis. Four mice were excluded for the following reasons: one mouse for low power and three mice due to a strong side preference.

#### Pharmacologic inhibition with intraperitoneal injections

C57BL/6J wild-type mice (for CCK-A inhibition, final *N* = 4, *N* = 3 male/1 female; for glutamate receptor inhibition, final *N* = 4, *N* = 2 male/2 female) underwent baseline choice assays as described in Choice assay. During the acclimation period, wild-type mice received intraperitoneal injections of PBS to acclimate them to the procedure. Once a stable sucrose preference was established, each mouse underwent two experimental conditions: drug and vehicle. The order of injection was counterbalanced. Testing occurred in our TSE PhenoMaster apparatus as described in the optogenetics section. For CCK-A inhibition, devazepide was delivered 2 mg kg^–1^ (10 μl g^–1^) dissolved in 5% DMSO in PBS. Devazepide or vehicle (5% DMSO in PBS) was injected intraperitoneally 30 min before the choice assay based on prior reports^30^. For glutamate receptor inhibition, KA/AP3 cocktail (15 ng kg^–1^ KA and 0.1 μg kg^–1^ AP3) or vehicle (1 M NaOH in PBS, pH 7.4) was delivered intraperitoneally (10 µl g^–1^) 10 min before the choice assay. Two mice were excluded from further study due to a strong side preference.

#### Pharmacologic inhibition with intraduodenal injections

C57BL/6J wild-type mice (final *N* = 4, *N* = 3 male/1 female) were implanted as described in the duodenal catheter surgery section and underwent baseline choice assays as described in Choice assay. Catheters were flushed with 0.05 ml of PBS daily and immediately before the 1-h food and water deprivation (dark onset). Testing occurred in our plexiglass cages with syringes (solution intake was measured manually every 5 min). Immediately before the 1-h test session, mice were attached to microrenathane tubing that delivered drug, vehicle or control solutions via an infusion pump (Fusion 200, Chemyx). The infusion pump was started, which delivered 0.4 ml of the appropriate solution over the 1-h test session (flow rate was 0.0066 ml min^–1^). PBS solutions were delivered intraintestinally during baseline tests. For glutamate receptor inhibition, KA/AP3 cocktail (15 ng KA and 0.1 μg AP3) or vehicle (1 M NaOH in PBS, pH 7.4) was delivered intraintestinally in 0.4 ml. Catheter placement was confirmed at the end of the study by visual inspection of the small intestine following the infusion of a dye solution through the catheter. One mouse was killed before the final baseline test out of concern for stability of the implant. However, the catheter was confirmed to be in the duodenum, and its data were included. One mouse was excluded from further study due to a strong side preference.

### One bottle assay

CckCRE_ChR2 mice (final *N* = 4 CckCRE_ChR2, *N* = 2 male/2 female; *N* = 4 littermate controls, *N* = 2 male/2 female) were acclimated to the PhenoMaster and implanted with flexible fiberoptic implants as described above (Gut fiberoptic implantation surgery*)*. Implanted mice were given free access to sucralose (15 mM) 24 h in the home cage to control for neophobia. During 24-h access, mice had ad libitum access to food and water, although water intake was negligible; implanted mice were not connected to patch cables during exposure. For each one-bottle assay, 5 min before the start of the dark cycle, access to food and water was closed, and mice were attached to the rotary joint patch cable (ThorLabs, RJPFF2) in the Phenomaster; mice remained in the home cage. At the start of the dark cycle, sucralose (15 mM) became available for free consumption for 1 h. Following each test session, mice were disconnected and given ad libitum access to food and water. The start of all test sessions was separated by at least 48 h.

Each mouse underwent two habituation conditions (sucralose (15 mM), no laser) followed by two experimental conditions: sucralose + 473 nm (activating) and sucralose + 532 nm (control). The order of experimental conditions was randomized across mice. During photostimulation conditions, the Phenomaster delivered a TTL pulse for laser stimulation based on intake as follows: for every 0.01 g of liquid consumed, the mice received 5 s of laser stimulation (5 V, 40 Hz, 20% duty cycle). Fiberoptic placement and power output was confirmed at the end of the study. Only mice who completed all tests and whose device had appropriate power/placement were included in the analysis. One mouse was excluded from the analysis due to a broken fiber in the lumen.

#### Pharmacological blockade

The test above was repeated in a separate group of CckCRE_ChR2 mice (*N* = 3, *N* = 1 male/2 female) with a slight modification. Thirty minutes before the onset of the dark cycle, food and water was removed. Mice received an injection of a cocktail of glutamate receptor antagonists (150 μg kg^–1^ KA and 1 mg kg^–1^ AP3, intraperitoneally) 25 min before access to sucralose, which coincided with the onset of the dark cycle. Mice had access to sucralose (15 mM) for 1 h.

### Statistics and reproducibility

We performed statistical analyses using JMP Pro Software (version 14, SAS), unless otherwise indicated. Data were evaluated for normality using a Q–Q plot. For normally distributed data, an ANOVA was used, and a Tukey’s honestly significant different post hoc testing was performed when applicable. For behavior studies, we used a repeated measures ANOVA to account for each subject and followed with post hoc paired Student’s *t*-tests. For data not normally distributed, means were evaluated by a Kruskal–Wallis test with non-parametric comparisons using the Wilcoxon method. For other studies, comments on statistical tests performed are included throughout the Methods and in figure legends. All error bars and shaded regions represent s.e.m. unless otherwise indicated. Sample size was not predetermined using power analyses. No statistical methods were used to predetermine sample sizes, but our sample sizes are similar to those reported in previous publications^33,44^. Standardized randomization was not performed for in vitro or in vivo experiments. All behavioral studies were counterbalanced across age and sex to control for variables including position in cage, order effect and handedness. Data collection and analysis were not performed blind to the conditions of the experiments.

### Reporting Summary

Further information on research design is available in the Nature Research Reporting Summary linked to this article.

## Online content

Any methods, additional references, Nature Research reporting summaries, source data, extended data, supplementary information, acknowledgements, peer review information; details of author contributions and competing interests; and statements of data and code availability are available at 10.1038/s41593-021-00982-7.

## Supplementary information

Reporting Summary

Supplementary Video 1 A flexible fiber for gut optogenetics. A conventional stiff silica fiber (left) punctures an agarose (1.5%) membrane, but the flexible PC/PMMA fiber (right) does not.

## Data Availability

The source data that support the findings of this study are available from the corresponding author upon request. The mm10 mouse reference genome is available from GENCODE vM23/Ensembl 98. Single-cell sequencing data sets are available on the NIH Gene Expression Omnibus database (GSE185173).

## Code availability

All custom code used for analysis in this paper is available from the corresponding author upon request.
