# Supplementary material for: The preference for sugar over sweetener depends on a gut sensor cell
Source: Nat Neurosci. 2022 Jan 13;25(2):191–200. doi: 10.1038/s41593-021-00982-7 (PMC8825280; doi:10.1038/s41593-021-00982-7)
Supplement: Supplementary file 1 — Reporting Summary [file 41593_2021_982_MOESM1_ESM.pdf]

nature portfolio

Corresponding author(s): Diego Bohórquez

Last updated by author(s): Nov 5, 2021

Reporting Summary

Nature Portfolio wishes to improve the reproducibility of the work that we publish. This form provides structure for consistency and transparency in reporting. For further information on Nature Portfolio policies, see our Editorial Policies and the Editorial Policy Checklist.

Statistics

For all statistical analyses, confirm that the following items are present in the figure legend, table legend, main text, or Methods section.

n/a	Confirmed	
☐	☒	The exact sample size (*n*) for each experimental group/condition, given as a discrete number and unit of measurement
☐	☒	A statement on whether measurements were taken from distinct samples or whether the same sample was measured repeatedly
☐	☒	The statistical test(s) used AND whether they are one- or two-sided<br>*Only common tests should be described solely by name; describe more complex techniques in the Methods section.*
☐	☒	A description of all covariates tested
☐	☒	A description of any assumptions or corrections, such as tests of normality and adjustment for multiple comparisons
☐	☒	A full description of the statistical parameters including central tendency (e.g. means) or other basic estimates (e.g. regression coefficient) AND variation (e.g. standard deviation) or associated estimates of uncertainty (e.g. confidence intervals)
☐	☒	For null hypothesis testing, the test statistic (e.g. *F*, *t*, *r*) with confidence intervals, effect sizes, degrees of freedom and *P* value noted<br>*Give P values as exact values whenever suitable.*
☒	☐	For Bayesian analysis, information on the choice of priors and Markov chain Monte Carlo settings
☒	☐	For hierarchical and complex designs, identification of the appropriate level for tests and full reporting of outcomes
☒	☐	Estimates of effect sizes (e.g. Cohen's *d*, Pearson's *r*), indicating how they were calculated

Our web collection on statistics for biologists contains articles on many of the points above.

Software and code

Policy information about availability of computer code

Data collection	Software used in data collection: Signal Express (NI; https://ni.com, version 16.0.1); pClamp (Axon Instruments; Software Version 10.7); ZEN 2 - Blue Edition (Zeiss; https://www.zeiss.com); PhenoMaster software (TSE Systems Inc.; software version 6.6.9)
Data analysis	Software used in data analysis: ImageJ/Fiji (Schneider et al 2012; https://imagej.nih.gov/ij/, version 2.3.0); MATLAB (MathWorks; https://www.mathworks.com, version R2021a 9.10); RStudio (R Consortium; https://www.r-project.org, version 2.10); JMP Pro (JMP from SAS; https://www.jmp.com, version 16); Spike Tailor (Mathworks; Kaelberer et al., 2018); Seurat (R Consortium; Stuart et al., 2019, version 3.1.0); Biomark- RealTime PCR Analysis (Fluidigm; https://www.fluidigm.com/software, PN 101-6793); Qlucore Omics Explorer (Qlucore; https://www.qlucore.com/omics-explorer, version 3.6)

For manuscripts utilizing custom algorithms or software that are central to the research but not yet described in published literature, software must be made available to editors and reviewers. We strongly encourage code deposition in a community repository (e.g. GitHub). See the Nature Portfolio guidelines for submitting code & software for further information.

Data

Policy information about availability of data

All manuscripts must include a data availability statement. This statement should provide the following information, where applicable:

- Accession codes, unique identifiers, or web links for publicly available datasets
- A description of any restrictions on data availability
- For clinical datasets or third party data, please ensure that the statement adheres to our policy

The source data that support the findings of this study are available from the corresponding author upon request. The mm10 mouse reference genome available from GENCODE vM23/Ensembl 98. Single cell sequencing datasets are available on the NIH GEO database (GSE185173).

Field-specific reporting

Please select the one below that is the best fit for your research. If you are not sure, read the appropriate sections before making your selection.

☒ Life sciences ☐ Behavioural & social sciences ☐ Ecological, evolutionary & environmental sciences

For a reference copy of the document with all sections, see nature.com/documents/nr-reporting-summary-flat.pdf

Life sciences study design

All studies must disclose on these points even when the disclosure is negative.

Sample size	No statistical methods were used to pre-determine sample sizes but our sample sizes are similar to those reported in previous publications studying ingestive behavior in mouse models (Han et al., Cell, 2018; Tan et al., Nature, 2020; Sclafani & Ackroff, Physiol. Behav., 2017).
Data exclusions	- For vagal nerve recordings: Throughout experiments, sucrose response was used as a positive control. For all nutrient and laser stimulation conditions, data were excluded if a stable sucrose response was not seen throughout the recording session.<br>- For single cell qPCR: All cells not meeting quality measures or having no detected transcripts for either housekeeping gene (Gapdh or Actb1) were excluded from analysis (48 positive cells, 24 negative cells were excluded).<br>- For calcium imaging and patch clamp electrophysiology: Each recording session concluded with 45 mM KCl as an activity control (KCl concentration was achieved by substituting for NaCl, and not an addition of more KCl). A response to KCl was defined as a ratio > 10% increase above baseline. Cells that did not reach this KCl threshold were not included in analyses.<br>- For in vivo calcium imaging of vagal nodose neurons: The predetermined exclusion criteria for neurons was a response to mannitol [300mM] because this meant the neuron was responsive to osmolarity instead of just sugar.<br>- For optogenetic behavior studies: Only mice who completed all tests and whose fiberoptic device had appropriate power/placement at completion were included in analysis. For two-bottle studies, mice that did not have a side preference were included.<br>- For pharmacologic behavior studies. Only mice who completed all tests, did not have a side preference, and whose catheter had appropriate placement and patency at completion were included in analysis.
Replication	- For vagal cuff experiments, the response to positive control sucrose was tested and replicated in between ligands to ensure within subject reproducibility. If the response changed substantially, the inclusion criterion was not met and therefore, the experiment was terminated. The vagal response to sucrose and sucralose was reproducible across at least 2 users.<br>- For in vitro calcium imaging and patch clamp electrophysiology, the response to stimuli within the same cell was not replicated due to limitations of cell viability with repeated applications. To ensure reproducibility, experiments were conducted across several sessions and included at least 2 independent biological replicates.<br>- For in vivo calcium imaging, each stimulant was perfused twice per mouse, leading to similar results. To ensure reproducibility, experiments were conducted across several sessions and included 4 independent biological replicates.<br>- For single cell qPCR, experiments were repeated three times using three biological replicates. The results of individual experiments were similar.<br>- For behavior experiments, response to optogenetic or pharmacologic inhibition was not replicated within subject because the durability of the implants was limited and multiple experiments were required from each mouse. To ensure reproducibility, mice across at least 3 litters were used for each experiment.
Randomization	Standardized randomization was not performed for in vitro or in vivo experiments. For vagal cuff experiments, sucrose 300mM was used as a positive control and the order of the subsequent ligands was random within each mouse. For in vitro calcium imaging experiments, in vivo calcium imaging experiments, and patch clamp electrophysiology, the order of the experimental stimuli was alternated to control for potential order effects. All behavioral studies were counterbalanced across age and sex to control for variables including position in cage, order effect, and handedness.
Blinding	Experimenters were not blinded to treatment condition, genotype, or outcome due to the need for the experimenter to give the desired test ligand or treatment.

Reporting for specific materials, systems and methods

We require information from authors about some types of materials, experimental systems and methods used in many studies. Here, indicate whether each material, system or method listed is relevant to your study. If you are not sure if a list item applies to your research, read the appropriate section before selecting a response.

Materials & experimental systems

n/a	Involved in the study
☐	☒ Antibodies
☒	☐ Eukaryotic cell lines
☒	☐ Palaeontology and archaeology
☐	☒ Animals and other organisms
☐	☒ Human research participants
☒	☐ Clinical data
☒	☐ Dual use research of concern

Methods

n/a	Involved in the study
☒	☐ ChIP-seq
☒	☐ Flow cytometry
☒	☐ MRI-based neuroimaging

Antibodies

Antibodies used

- For immunohistochemistry: Anti-SGLT1 antibody (host = rabbit) (Abcam; Cat#ab14686); Anti-GFP antibody (host = chiken) (Abcam; Cat#ab13970); Alexa Fluor 488 AffiniPure F(ab') Fragment Donkey Anti-Rabbit IgG (H+L) (Jackson ImmunoResearch; Cat#711-546-152; RRID#AB_2340619); Cy3 AffiniPure F(ab') Fragment Donkey Anti-Rabbit IgG (H+L) (Jackson ImmunoResearch; Cat#711-166-152; RRID#AB_2313568); Alexa Fluor 488 AffiniPure F(ab') Fragment Donkey Anti-Chicken IgG (H+L) (Jackson ImmunoResearch; Cat#703-546-155; RRID#AB_2340376)
- For in situ hybridization: All probes were purchased from ACD including Mm-Cck (cat# 402278), Mm-Slc5a1 (cat# 468888), and Mm-Tas1r3 (cat# 515431)

Validation

Anti-SGLT1 antibody was commercially validated in human enterocytes, heart and skeletal muscle tissues. The antibody was then validated for this study in murine enterocytes as positive control. Anti-GFP antibody was commercially validated in mouse tissue against recombinant fragment.

Animals and other organisms

Policy information about studies involving animals; ARRIVE guidelines recommended for reporting animal research

Laboratory animals

Male and female adult mice aged 6-20 weeks were used in all experiments. Mice were group housed in Duke University's Division of Laboratory Animal Resources, where they were kept on a 12-hour light-dark cycle (0700-1900) with access to water and standard mouse chow (Purina 5001) ad-libitum, unless otherwise indicated in the manuscript Methods. The facility maintained an ambient temperature of 18-23°C and humidity of 40-60%. Mice used were: C57BL/6J (wild-type) (Jackson Lab; Stock #000664); Swiss Webster (wild-type) (Charles River; Stock #024); CckGFP (background = Swiss Webster) (Rodger Liddle, M.D.; Wang et al., 2010); CckCRE (background = C57BL/6J) (Jackson Lab; Stock #012706); Neurod1CRE (background = C57BL/6J) (Jackson Lab; Stock #028364); LSL_tdTomato (background = C57BL/6J) (Jackson Lab; Stock #007914); LSL_Halo-YFP (background = C57BL/6J) (Jackson Lab; Stock #014539); LSL_ChR2-tdTomato (background = C57BL/6J) (Jackson Lab; Stock #012567); LSL_Salsa6f (background = C57BL/6J) (Jackson Lab; Stock #031968)

Wild animals

No wild animals were used in the study.

Field-collected samples

No field-collected samples were used in the study.

Ethics oversight

All experiments on mice were performed following approval by the Institutional Animal Care and Use Committee at Duke University Medical Center under the protocol A280-18-12.

Note that full information on the approval of the study protocol must also be provided in the manuscript.

Human research participants

Policy information about studies involving human research participants

Population characteristics

Human samples were received de-identified. Patient characteristics were unknown.

Recruitment

Participants were recruited through the Duke University Medical Center Biorepository and Precision Pathology Center (BRPC) under the Institutional Review Board (IRB) protocol Pro00035974 via anonymous tissue release.

Ethics oversight

Human duodenal samples were obtained from the Duke University Medical Center Biorepository and Precision Pathology Center (BRPC) under the Institutional Review Board (IRB) protocol Pro00035974 via anonymous tissue release. Per this protocol, informed consent was obtained from all study participants. All samples were deidentified and all links to additional patient information were broken prior to receipt of fresh surgical specimens.

Note that full information on the approval of the study protocol must also be provided in the manuscript.
